# Dopaminergic signaling regulates microglial surveillance and adolescent plasticity in the mouse frontal cortex

**DOI:** 10.1038/s41467-025-63314-4

**Published:** 2025-08-26

**Authors:** Rianne Stowell, Kuan Hong Wang

**Affiliations:** Department of Neuroscience, Del Monte Institute for Neuroscience, University of Rochester Medical Center, Rochester, NY USA

**Keywords:** Neural circuits, Microglia

## Abstract

Adolescence is a sensitive period for frontal cortical development and cognitive maturation, marked by heightened structural plasticity in the dopaminergic (DA) mesofrontal circuit. However, the cellular and molecular mechanisms underlying this plasticity remain unclear. Here, we show that microglia, the brain’s innate immune cells, are highly responsive to mesofrontal DA signaling during adolescence. Longitudinal in vivo two-photon imaging in mice reveals that frontal cortical microglia increase their surveillance of the parenchyma and DA axonal boutons following rewarding experiences or optogenetic stimulation of DA axons. Microglial contacts with DA axons consistently precede bouton formation, and microglia-bouton interactions are regulated by D1- and D2-type DA receptors in adolescence and adulthood. Furthermore, microglial purinergic receptor P2RY12 signaling is necessary for enhanced microglial surveillance and DA bouton formation during adolescence. These results uncover bidirectional interactions between DA signaling and microglial surveillance that drive adolescent frontal plasticity and identify potential targets for restoring plasticity in adulthood.

## Abstract (web summary)

Adolescence is a critical period for brain development, marked by heightened plasticity in the frontal dopamine circuit. Here, the authors show in mice that adolescent microglia respond to dopamine with increased surveillance to promote dopaminergic innervation.

## Introduction

Adolescence is increasingly viewed as a sensitive period for the structural and functional development of the frontal cortex^1,2^. Psychiatric disorders, such as schizophrenia and attention deficit hyperactivity disorder (ADHD), frequently manifest in adolescence^3^. This coincides with the maturation of the dopaminergic (DA) mesofrontal circuit, which connects the midbrain motivation center to the frontal cortical cognitive control center^4–6^. Importantly, disruptions in the development and function of the frontal cortical dopamine system are key characteristics of neurodevelopmental psychiatric disorders^7,8^. Despite our long-standing recognition of the disease relevance of frontal DA dysfunction, little is known about the cellular mechanisms involved in the adolescent plasticity of frontal DA circuits.

The adolescent mesofrontal circuit is malleable in response to changes in behavioral experience and DA activity^9,10^. Frontal DA axons exhibit robust outgrowth of boutons, the main release sites for DA^6,11^, in response to rewarding behavioral experiences such as wheel-running in mice^9^. Furthermore, directly increasing the phasic activity of ventral tegmental area (VTA) DA neurons, a pattern of activity typically associated with reward^12^, induces frontal DA bouton formation, as well as functional changes in frontal cortical activity and psychomotor behavior^9^. Adolescent stimulation of VTA DA neurons or frontal DA projections to drive mesofrontal plasticity rescues frontal circuit and behavioral deficits in multiple genetic mouse models of neurodevelopmental disruption^13^. However, mesofrontal DA circuit plasticity is unique to adolescence, as phasic VTA stimulation alone does not elicit these effects in adult animals^9^, suggesting that the adolescent frontal cortex is uniquely permissive of activity-dependent changes. Notably, adult plasticity can be reinstated when VTA stimulation is paired with pharmacological blockade of the D2-type DA receptor (D2R)^9^. However, it remains unknown what cellular players are engaged in mesofrontal plasticity during adolescence and the reinstating of this plasticity in adulthood.

In pursuit of candidate regulators of mesofrontal DA plasticity, we investigated the adolescent microglial response to DA signaling. Beyond their roles in pathological conditions, microglia, the innate immune cells of the central nervous system (CNS), can also modulate neural circuit development and activity-dependent plasticity^14–18^. Recently, microglia have been recognized as a heterogeneous population of cells with regional specificity in both gene expression and responsiveness to CNS signals^19^. The differences between subcortical and cortical microglia have been examined through RNA sequencing studies, but the developmental and spatial diversity of microglia across cortical regions remains unexplored^20^. While microglia respond to a few neurotransmitters such as norepinephrine^15,21^, GABA^22^, glutamate^23^, and ATP^24^ in several brain regions, it is unknown if microglia respond to DA signals in vivo. Previous in vitro work suggests that microglia express DA receptors and can respond to DA signaling^25^. Recent studies in the basal ganglia have found that microglia exhibit region-specific phenotypes^26^ and influence neuronal DA receptor expression in the adolescent nucleus accumbens^18^. Spatial transcriptomic data confirm that a subset of frontal cortical microglia express D1R and D2R^27^. However, previous research has not investigated whether microglia respond to DA in vivo or interact with the frontal DA circuit. Thus, evaluating microglia as a potential cellular player in frontal DA circuit plasticity may provide key insights into plasticity mechanisms and inform future therapeutic strategies.

Here, we investigated if frontal cortical microglia respond to DA signals from the mesofrontal circuit. We show that in vivo adolescent microglia respond to the activity of the mesofrontal circuit elicited by both wheel running and direct phasic activation of the mesofrontal DA axons. Our findings depict a biphasic microglial response to phasic mesofrontal activation, characterized by an initial retraction of microglial processes during stimulation and a subsequent sustained increase in parenchymal surveillance, which includes enhanced contact with axonal elements. During axonal surveillance, microglial contacts along the axon backbone precede the formation of new boutons. Furthermore, perturbing DA signaling through pharmacological manipulation of either D1 or D2 receptors can eliminate the biphasic response of frontal cortical microglia to phasic DA activity and block new bouton formation. Moreover, reinstating plasticity in adult mice by D2 antagonism results in an adolescent-like microglial DA response and increased surveillance of DA boutons. Finally, we demonstrate that microglial purinergic receptor P2RY12 signaling is necessary for both the biphasic microglial response to DA axon stimulation and activity-dependent axonal plasticity. Our work shows that adolescent frontal cortical microglia respond rapidly to mesofrontal DA signaling and that their dynamics are tightly coupled to both phasic mesofrontal activity and subsequent DA bouton outgrowth in the frontal cortex.

## Results

### Adolescent wheel running promotes DA bouton formation and microglial process outgrowth in the frontal cortex

**Fig. 1:**
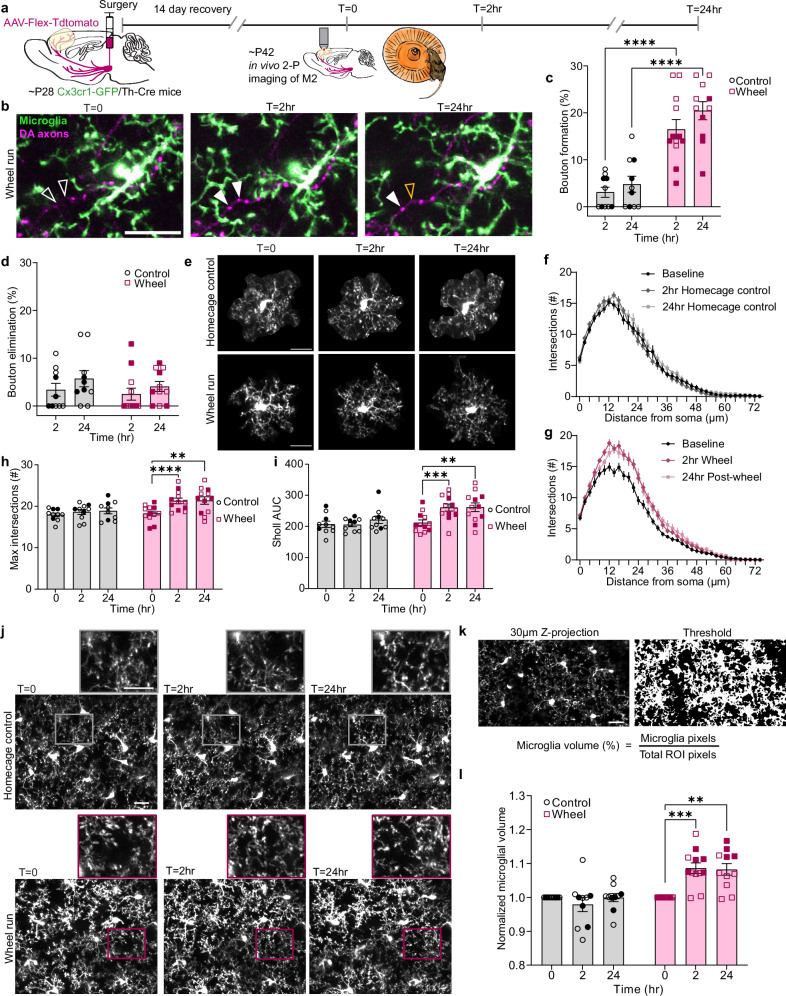
Adolescent wheel running promotes DA bouton formation and microglial process outgrowth. **a** Timeline of surgical and imaging procedures. **b** Example of microglia (green) and axons (magenta) at *t* = 0 (pre-run), after 2 hr of wheel running, and 24 hr later. Hollow arrowheads in *t *= 0 denote areas where new boutons form post-wheel run (solid arrowheads), with the hollow orange arrowhead denoting a lost bouton at *t* = 24 hr. **c** 2 hrs of wheel running promotes formation of new boutons (n: control = 10, wheel = 12 mice Mixed-effects model, Fixed effects [type III], Run status *p* < 0.0001, F (1,20) = 36.84, Šídák’s multiple comparisons Control v. Wheel, 2hr *p* < 0.0001 & 24 hr *p *< 0.0001). **d** Wheel running does not affect the rate of bouton elimination (n: control = 10, wheel = 12 mice, Mixed-effects model, Fixed effects [type III], Run status *p* = 0.5080, F(1,20) = 0.5453, Šídák’s multiple comparisons test Control v. Wheel, 2 hr *p* = 0.8682 & 24 hr *p* = 0.7674). **e** Example of individual microglia used in Sholl analysis. **f** Sholl profiles of home cage control microglia. **g** Sholl profiles of wheel run microglia. **h** Wheel running increases the microglial maximum number of intersections (n: control = 10, wheel = 12 Two-way RM ANOVA Time x Run status *p* = 0.0355 F(1.67,33.37) = 3.949 Dunnett’s multiple comparisons Wheel 0 v. Wheel 2 hr *p* < 0.0001 Wheel 0 v. Wheel 24 hr *p* = 0.0065). **i** Wheel running increases AUC of microglial Sholl profiles (n: control = 10, wheel = 12 Two-way RM ANOVA Time x Run status *p* = 0.0009 F(2,40) = 8.431 Dunnett’s multiple comparisons Wheel 0 v. Wheel 2 hr *p* = 0.0002 Wheel 0 v. Wheel 24 hr *p* = 0.0012). **j** Example 30 µm z-projections of microglia. Rectangle pop-outs denote representative areas of microglial process volume. **k** Visual representation of microglial volume analysis. **l** Wheel running increases microglial volume of the parenchyma. (n: control = 10, wheel = 12 mice, Mixed-effects model, Fixed effects [type III], Time x Run status *p* < 0.0001, F(2,39) = 12.17, Tukey’s multiple comparisons wheel 0 v. 2 hr *p* = 0.0004, 0 v. 24 hr *p* = 0.0017). Scale bars 20 µm. Multiple comparisons are two-sided. Graphs show mean ± S.E.M **p < 0.01, ***p < 0.005, ****p < 0.0001. Individual points represent individual animals with females as hollow symbols and males as solid symbols. Source data are provided as a source data file.

In order to determine if microglia respond to wheel-running induced mesofrontal plasticity, we utilized Cx3cr1^GFP^ mice crossed to a Th-Cre line so that microglia would be labeled with GFP and DA projections from the VTA could be specifically labeled through AAV-CAG-FLEX-tdTomato injected into the VTA^9,13,15^(Fig. 1a). We used Th-Cre rather than DAT-Cre as mesofrontal DA neurons have much stronger TH expression but little DAT expression^28,29^, which limits the utility of the DAT-Cre line for our target DA neuronal population. To verify Th-Cre was the appropriate choice, we confirmed that our virus transduction was limited to the VTA injection site (Supplemental Fig. 1a, b). We also evaluated DAT-Cre/Ai14 mice and found that there was a lack of DA axon labeling in the frontal cortex as well as spurious ectopic neuronal labeling^30^ (Supplemental Fig. 1c, d), suggesting that these mice would not be efficacious for our experimental purposes.

For all experiments, we used a chronic cranial window approach to allow for repeated imaging and to avoid the potential confound of the effects of anesthesia on microglial dynamics^15^. We selected the M2 area of the frontal cortex for imaging as it is optically accessible without the need for an invasive approach, which would disrupt microglia dynamics. In addition, this area receives robust dopaminergic innervation for in vivo imaging^4,9,31^. Our mice were imaged in the middle of adolescence between P35-P49^32^ when the mesofrontal circuit is still undergoing maturation^4^ and remains plastic^9^. We also confirmed that chronic cranial window preparations did not impact wheel running behavior (Supplementary Fig. 2a).

We found that after two hours of wheel running, both male and female mice showed increased rates of bouton formation and that this increase in boutons was maintained at 24 h (Fig. 1b, c, Mixed-effects model, Fixed effects [type III], Run status *p* < 0.0001). The increased bouton formation was not accompanied by a change in elimination (Fig. 1d Mixed-effects model, Fixed effects [type III], Run status, *p* = 0.4069), demonstrating that in both male and female adolescent mice wheel running generates a net gain in DA boutons. Although both male and female mice had increased bouton formation post wheel running, further analysis found that female mice had significantly higher bouton formation rates than males (Mixed-effects model, Fixed effects [type III], Sex x Run status *p* = 0.0221). We did not find a significant difference between male and female run distance (Supplementary Fig. 2b), nor a significant correlation between run distance and bouton formation rate (Supplementary Fig. 2c).

On the same timescale as the bouton changes, we found significant changes in microglial arborization. We first assayed microglial morphology using Sholl analysis (Fig. 1e–g Sholl curves representing an average of 3 microglia per time point per animal) and observed a clear increase in arborization at both 2 h and 24 h post-wheel running. This was quantified as a significant increase in both the maximum number of intersections and the area under the curve (AUC) of the Sholl profiles (Fig. 1h, i Two-way RM ANOVA Time x Run status *p* = 0.0229; Two-way RM ANOVA Time x Run status *p* = 0.0009). We next assessed how collectively these individual microglial morphological changes alter the overall volume of microglial processes present in the region of interest (ROI). We found that with the increased microglial arborization post-wheel running, the microglial processes appear to be more extensively distributed throughout the parenchyma (Fig. 1j). To quantify this visual observation, we measured microglial volume by z-projecting the maximum intensity from a 30 µm image stack onto a 2D plane, followed by thresholding to determine the percentage of microglia pixels relative to the total pixels in the field of view (Fig. 1k). When normalized to baseline (time 0), microglial volume shows a robust increase at both 2- and 24 h post-wheel running, positively correlating with the results of our Sholl analysis (Fig. 1l. Mixed-effects model, Fixed effects [type III], Time x Run status *p* < 0.0001; Supplementary Fig. 2d, e). Thus, 2 h of wheel running, which is known to drive phasic VTA activity^33^, is sufficient to generate both DA bouton formation and concomitant increases in microglial arborization.

Our previous work demonstrated that mesofrontal plasticity from wheel running was unique to adolescence, and that adult mice did not show a significant increase in DA bouton formation after 2 h of wheel running^9^. Our adult mice ran significantly more in the 2 h than the adolescent mice (Supplementary Fig. 3a), but neither male nor female adult mice showed any changes in bouton formation or elimination after wheel running (Supplementary Fig. 3b, c). The adult mice also showed no changes in microglial arborization (Supplementary Fig. 3d, e), suggesting that the effect of wheel-running on microglia in adolescence was not simply a product of exercise, particularly because the adults actually ran further than the adolescents.

To further test the specificity of wheel-running induced adolescent microglia changes to the frontal cortex, we evaluated mice with windows over the primary visual cortex (V1), which lacks significant DA innervation as compared to M2^34^ (Supplementary Fig. 3f). We found that in V1, microglia did not respond to wheel running (Supplementary Fig. 3g, h) Thus, the effect of wheel-running on microglia is specific to adolescence and the cortical region receiving major DA input from the VTA.

### Microglial surveillance decreases during DA axon stimulation and then increases post-stimulation in the adolescent frontal cortex

After observing the parallel changes in DA boutons and microglia in the frontal cortex induced by wheel running, we wanted to further determine if microglia respond to direct changes in DA neuron activity. To this end, we injected AAV2/9-CAG-Flex-ChR2-tdTomato into the VTA of Th-Cre mice to allow for optogenetic stimulation of DA pathways. With direct light stimulation of mesofrontal DA axons over the cranial window, we confirmed the efficacy of this stimulation method by conducting two-photon calcium imaging of the cortical neural response to phasic DA stimulation^13,35^ (Supplementary Fig. 4).

**Fig. 2:**
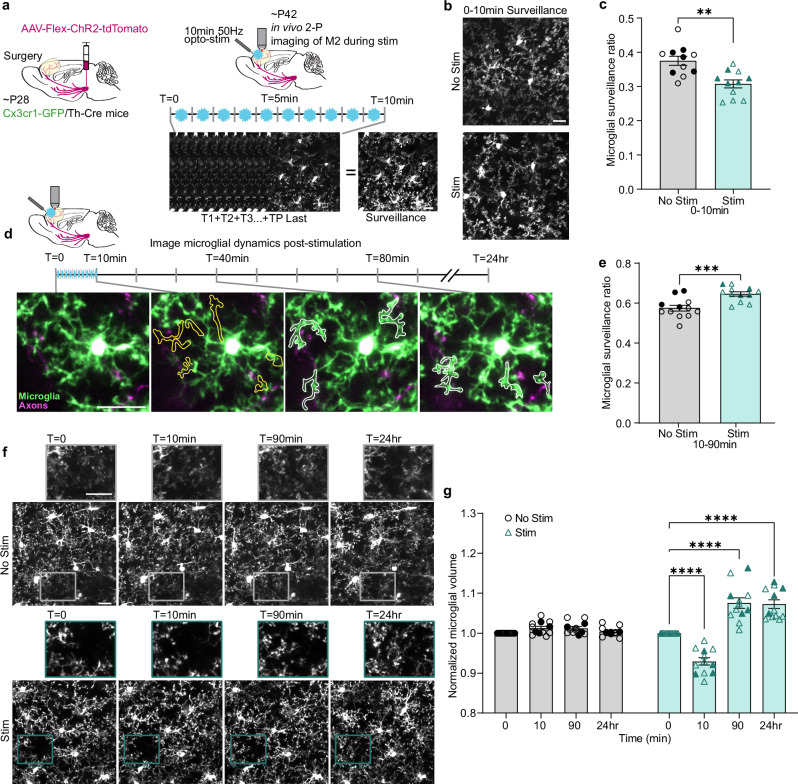
Microglia exhibit a biphasic response to optogenetic stimulation of DA axons. **a** Diagram of stimulation and image collection parameters. **b** Example microglial surveillance with and without optogenetic stimulation. **c** Microglial surveillance is reduced during phasic stimulation of the mesofrontal DA axons (*n* = 11 mice, two-tailed paired *t* test, *p* = 0.0015, t(10) = 4.324, effect size = 1.303). **d** Example of individual microglial process retraction and extension during imaging. Immediately after stimulation, microglial processes occupy less parenchyma (retracted processes outlined in yellow). Over the course of 90 min, microglia extend processes (white outline marking process outgrowth) toward axons (magenta). **e** After optogenetic stimulation, microglia increase surveillance of the parenchyma (*n* = 12 mice, two-tailed paired *t* test, *p* = 0.0003, t(11) = 5.213, effect size = 1.505). **f** Representative images of microglial volume changes pre-stim (*T* = 0), immediately after Stim (*T* = 10 min), 90 min later, and 24 hr after Stim, as well as paired No Stim timepoints (rectangular pop-outs highlight changes in microglial processes). **g** DA axon stimulation drives a biphasic microglial volume change with an initial reduction followed by a prolonged increase in microglial volume (*n* = 12 mice, two-way repeated measures ANOVA time x stimulation *p* < 0.0001, F(3,33) = 47.02, Holm-Šídák’s multiple comparisons Stim 0 v. 10 min *p *< 0.0001, 0 v. 90 min *p* < 0.0001, & 0 v. 24 hr *p* < 0.0001). Scale bars 20 µm. Multiple comparisons are two-sided. Graphs show mean ± S.E.M **p < 0.01, ***p < 0.005, ****p < 0.0001. No stim and stim experiments were conducted within the same animals. Individual points represent individual animals with females as hollow symbols and males as solid symbols. Source data are provided as a source data file.

Based on the tight temporal control of stimuli enabled by our optogenetic approach, we characterized the time course of the microglial response to phasic DA axon stimulation (473 nm, 20 mW output, 50 Hz pulse train, 3 ms/pulse, 10 pulses/train, 1 train/min for 10 min) by imaging microglia every minute between our pulses of light stimulation (Fig. 2a). Using these sequential images, we assayed microglial surveillance, a composite measure of microglial dynamics which reflects both morphological and motility changes^15,16^. By collapsing images across time, we can quantify all the areas microglia surveyed within our sequence of images (Fig. 2a). We found that in response to DA axon activation, microglia reduced their surveillance of the surrounding parenchyma as compared to their baseline surveillance without stimulation (Fig. 2b, c paired *t* test, *p* = 0.0015, Supplemental Videos 1 and 2). Upon cessation of stimulation (from 10 to 90 min after the start of the first stimulation), we found that microglial surveillance rapidly recovered and significantly surpassed the level of surveillance in unstimulated animals (Fig. 2d, e paired *t* test, *p* = 0.0003, Supplemental Videos 3 and 4).

**Fig. 3:**
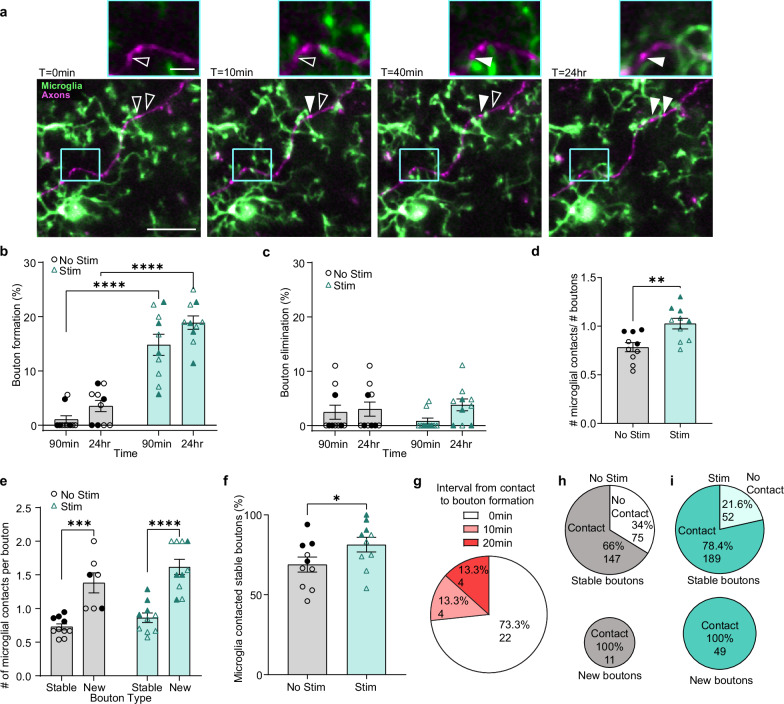
Microglia contact the axonal sites for newly formed boutons and increase surveillance of stable boutons after adolescent phasic DA axon activity. **a** Representative images of bouton formation (hollow arrowheads represent sites of future boutons, solid arrowheads denote a newly formed bouton). Rectangular pop-outs highlight microglial processes contacting axons prior to new bouton formation. **b** Phasic stimulation of DA axons drives bouton formation (*n* = 10 mice, two-way repeated measures ANOVA stimulation *p* < 0.0001, F(1,18) = 128.6, Šídák’s multiple comparisons No Stim v. Stim 90 min *p* < 0.0001, 24 hr *p* < 0.0001). **c** Phasic stimulation of DA axons does not drive bouton elimination (*n* = 10 mice, two-way repeated measures ANOVA, stimulation *p* = 0.8194, F(1,18) = 0.05369, Šídák’s multiple comparisons test No Stim v. Stim 90 min *p* = 0.5825, 24 hr *p* = 0.8121). **d** Microglia contact boutons more frequently after phasic DA axon stimulation (*n* = 10 mice, two-tailed paired *t* test, *p* = 0.0049 t(9) = 3.702). **e** Microglia make more frequent contacts with newly formed than stable boutons (*n* = 10 mice, Mixed-effects model, Fixed effects [type III], Stability *p* < 0.0001, F(1,9) = 45.21, Šídák’s multiple comparisons Stable v. New No stim *p* = 0.0001, Opto-stim *p* = 0.0008). **f** After phasic DA axon stimulation, microglia contact a larger proportion of stable boutons (*n* = 10 mice, two-tailed paired *t* test, *p* = 0.0329, t(9) = 2.518). **g** Pie chart representation of new boutons formed within the 90 min imaging period post-stimulation, sub-grouped by the time from microglial contact to bouton formation. 0 min indicates microglial processes were still in contact at the moment of formation. 2 boutons were excluded as they formed during the stimulation period and could not be assayed for contact. **h**,** i** Pie charts showing pooled percentage (%) and number of stable and new boutons contacted by microglia in no stimulation (**h)** and stimulated (**i)** conditions. A higher % of stable boutons are contacted by microglia after stimulation. New boutons are always contacted by microglia and are more abundant after stimulation (illustrated by the pie chart size difference). Scale bars 20 µm. Multiple comparisons are two-sided. Graphs show mean ± S.E.M *p < 0.05, **p < 0.01, ***p < 0.005, ****p ≤ 0.0001. No Stim and Stim experiments were conducted within the same animals. Individual points represent individual animals with females as hollow symbols and males as solid symbols. Source data are provided as a source data file.

We then further assessed the changes over the entire course of stimulation and imaging by calculating microglial volume at 0 min, 10 min, 90 min, and 24 h. We chose to analyze volume, as it was an efficient and effective method to capture increased arborization in the wheel running dataset, which positively correlated with our Sholl results (Supplementary Fig. 2d, e). By examining volume at these individual time points, we observed an initial dip in volume from 0 to 10 min, corresponding with the stimulation period, followed by a significant increase in volume by 90 min, which was sustained to 24 h (Fig. 3f, g two-way repeated measures ANOVA time x stimulation *p* < 0.0001). This was not an effect of the light exposure itself, as a cohort expressing only tdTomato while receiving light stimulation did not show these responses (Supplemental Fig. 5a–c).

Comparing our optogenetic stimulation to our wheel running cohort, we see a parallel in the post-treatment microglial response pattern—after phasic stimulation of M2 DA axons or wheel running, microglia undergo process elaboration and maintain this increased parenchymal volume for 24 h post-stimulation. This increase in microglial volume in both wheel running and optogenetic stimulation conditions coincides with the time period when new DA boutons formed with wheel running. Thus, we next evaluated what relationship increased microglial surveillance may have to DA bouton plasticity with the optogenetic approach.

### Microglia contact the axonal sites for newly formed boutons and increase surveillance of stable boutons after adolescent DA axon stimulation

We first confirmed that, as in the wheel running paradigm, optogenetic phasic DA axon stimulation resulted in increased bouton formation at 2 h, which was sustained out to 24 h without a concomitant change in elimination (Fig. 3a–c; formation two-way repeated measures ANOVA stimulation *p* < 0.0001; elimination two-way repeated measures ANOVA, stimulation, *p* = 0.8194). To track microglial contacts with boutons, we identified stable boutons (boutons present throughout all the imaging time points), new boutons (boutons appearing along the axon backbone after *t* = 0), and eliminated boutons (boutons present at *t* = 0 but disappearing prior to or by 24 h). For new or eliminated boutons, microglial contacts with the axon backbone at the site of future bouton formation or post-bouton elimination were counted in the total contacts for each category of boutons.

Analysis of microglial contacts with DA boutons revealed a tight coupling between microglial dynamics and bouton plasticity. During the 90 min post-stimulation imaging period, there was a significant increase in microglial contacts with DA boutons (Fig. 3d two-tailed paired *t* test, *p* = 0.0049, Supplemental Video 5). When bouton contact data was subdivided based on bouton stability, we found that microglia make more contacts per new bouton than per stable bouton (Fig. 3e Mixed-effects model, Fixed effects [type III], Stability *p* < 0.0001). This pattern was present in both unstimulated and stimulated conditions; however, it is essential to note that there were far more new boutons in the stimulated (49 boutons) versus the unstimulated condition (11 boutons). Thus, newly formed boutons contribute to the overall increase in microglial contacts per bouton after optogenetic stimulation. This suggests that optogenetic stimulation amplifies the natural recruitment of microglial processes to the sites of new bouton formation. In addition to the frequent contacts with the sites of new boutons, microglia contacted a larger proportion of the stable boutons post-stimulation (Fig. 3f, paired *t* test, *p* = 0.0329). This suggests a specific change in the pattern of microglial interactions with stable boutons after optogenetic stimulation.

A final, and surprising finding is that microglial contact of the axon backbone precedes the formation of new boutons. In all cases where we could observe the axon backbone before new bouton formation, we found microglial processes contacting the axonal sites prior to the appearance of new boutons (Fig. 3a, g). In the case of the 29 new boutons which formed within the 90 min imaging period post-stimulation, 21 boutons first appeared while the microglial process was still in contact with the axon, whereas the rest appeared within 20 min after microglia contact (Fig. 3g). There were only 2 new boutons which formed within the 90 min imaging period in the unstimulated condition, and these also received microglial contact prior to bouton formation. Another population of boutons were detected only at the 24 h sampling time, so their temporal relationship with microglia contact could not be determined precisely due to the impracticality of imaging throughout the 24 h interval. In both unstimulated and stimulated conditions, all new boutons were preceded by microglial axon backbone contact (Fig. 3h, i).

While there were few eliminated boutons in either condition (unstimulated 7 boutons and stimulated 8 boutons), we saw that like newly formed boutons, eliminated boutons received more frequent contacts than stable boutons and eliminated boutons were always contacted by microglial processes prior to elimination (Supplementary Fig. 6). We also confirmed that light stimulation in no-opsin control animals did not show any changes in bouton dynamics or shifts in microglial contacts with boutons (Supplementary Fig. 7). Taken together, our observations suggest that microglia are highly sensitive to changes in DA activity, and there is a compelling connection between microglial contact and structural changes at the axon.

### Pharmacological manipulation of DA receptor signaling disrupts the biphasic microglial response to DA axon stimulation in adolescence

**Fig. 4:**
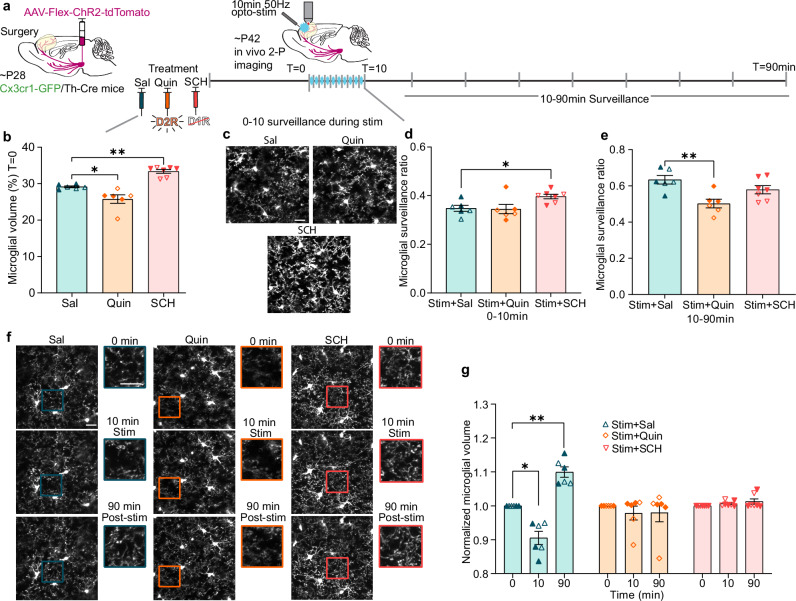
Manipulating DA receptor signaling disrupts adolescent microglial dynamics. **a** Timeline of dosing and imaging. **b** D2R agonism (Quinpirole 1 mg/kg, i.p. Quin) decreases baseline microglial parenchyma volume while D1R antagonism (SCH 23390 1 mg/kg, i.p. SCH) increases baseline volume (n: Sal = 6, Quin = 6, SCH = 7 mice, one-way ANOVA *p* < 0.0001, F(2,16) = 28.21, Dunnett’s multiple comparisons test, Sal v. Quin *p *= 0.0111 Effect size=1.177, Sal v. SCH* p* = 0.0013 Effect size=3.1165). **c** Representative images of microglial surveillance during phasic DA axon stimulation. **d** SCH prevents the expected decrease in microglial surveillance from 0–10 min during DA axon stimulation (n: Sal = 6, Quin = 6, SCH = 7 mice, one-way ANOVA *p* = 0.0270, F(2,16) = 4.565, Dunnett’s multiple comparisons Stim+Sal v. Stim + SCH *p* = 0.0426). **e** Quin prevents increased surveillance from 10–90 min post-DA axon stimulation (n: Sal = 6, Quin = 6, SCH = 7 mice, one-way ANOVA *p* = 0.0051, F(2,16) = 7.490, Dunnett’s multiple comparisons test, Stim + Sal v. Stim + Quin *p* = 0.0027). **f** Representative images of microglial volume. Rectangle pop-outs denote magnified areas. **g** Both Quin and SCH prevent the dynamic changes in microglial volume which accompany phasic DA axon stimulation (n: Sal = 6, Quin = 6, SCH = 7 mice, two-way repeated measures ANOVA, Time x Treatment *p* < 0.0001, F(4,32) = 20.33, Tukey’s multiple comparisons Stim + Sal 0 v. 10 min *p* = 0.0103, 0 v. 90 min *p* = 0.0030, 10 v. 90 min *p* = 0.0037). Scale bars 20 µm. Multiple comparisons are two-sided. Graphs show mean ± S.E.M *p < 0.05, **p < 0.01, ***p < 0.005, ****p < 0.0001. Individual points represent individual animals with females as hollow symbols and males as solid symbols. Source data are provided as a source data file.

In order to further assess the role of DA signaling in the relationship between microglial contacts and DA bouton formation, we altered either D1 or D2 receptor function pharmacologically prior to our stimulation paradigm (Fig. 4a). Our previous work showed that administering the D2R agonist quinpirole (Quin 1 mg/kg i.p.) was sufficient to block adolescent mesofrontal plasticity, implicating D2R stimulation as a negative regulator of mesofrontal plasticity^9^. Thus, we decided to test if this D2R manipulation would also produce changes in microglial dynamics. Additionally, because D1 and D2 receptors generate opposing G-protein coupled downstream signals^36^, we hypothesized that D1R signaling may then be a potential positive driver of mesofrontal plasticity and microglial dynamics. We used the D1R antagonist SCH23390 (SCH 1 mg/kg i.p.) to reduce D1R activity and test its involvement in microglia dynamics.

We found that administration of Quin significantly reduced baseline microglial arborization as measured by volume, while SCH significantly increased volume (Fig. 4b one-way ANOVA *p* < 0.0001). From 0–10 min during DA axon stimulation, microglia surveillance in the saline (Sal) control group decreased to a level similar to what we observed in our initial experiments without any saline or drug injection (Fig. 2c). The Quin treated group showed a similar level of surveillance to the Sal control, but the SCH treated group maintained a significantly higher level of surveillance and did not respond to DA axon stimulation (Fig. 4c, d one-way ANOVA *p* = 0.0270, Supplemental videos 6–8). In our initial optogenetic stimulation experiments, we also saw that 10–90 min post-stimulation microglial surveillance increased (Fig. 2e). The Sal control group followed this pattern, achieving surveillance levels comparable to our first cohort (Fig. 4e). The SCH-treated mice also had high microglial surveillance post-stimulation, but the Quin-treated mice had decreased microglial surveillance (Fig. 4e one-way ANOVA *p* = 0.0051, Supplemental Videos 9–11). To better parse how Quin and SCH were impacting the biphasic microglial response to adolescent DA axon stimulation, we evaluated microglial volume at 0, 10, and 90 min. We found that, like our previous stimulation cohort, the Sal-treated control animals showed a reduction at 10 min when stimulation had just ended and then a rebound in arborization by 90 min post-stimulation. In comparison, there was no dynamic change in either Quin or SCH treated groups (Fig. 4f, g two-way repeated measures ANOVA, Time x Treatment *p* < 0.0001).

Taken together, these results suggest that the pharmacological manipulation of D1R or D2R receptors was sufficient to cause a sustained change in microglial arborization and surveillance. D2R agonism induced microglial process retraction, rendering them in a state similar to that observed during DA axon stimulation, and precluded further dynamic surveillance changes in response to DA stimulation. On the other hand, D1R antagonism increased microglial arborization and kept them in this expanded state with enhanced surveillance, but precluded further dynamic responses to DA axon stimulation. Thus, D1R and D2R signaling may both be involved in the biphasic microglial response to DA axon stimulation. Recent spatial transcriptomic studies have revealed that DRs are expressed in frontal cortical microglia, in addition to neurons^27,37^ (Supplementary Fig. 8), suggesting that the observed effects of DR manipulation may arise from direct microglial DR signaling, interactions between microglia and DA-receptive neurons, or both.

### Altering DA receptor signaling disrupts adolescent mesofrontal axon plasticity and reduces microglial contacts with DA boutons

**Fig. 5:**
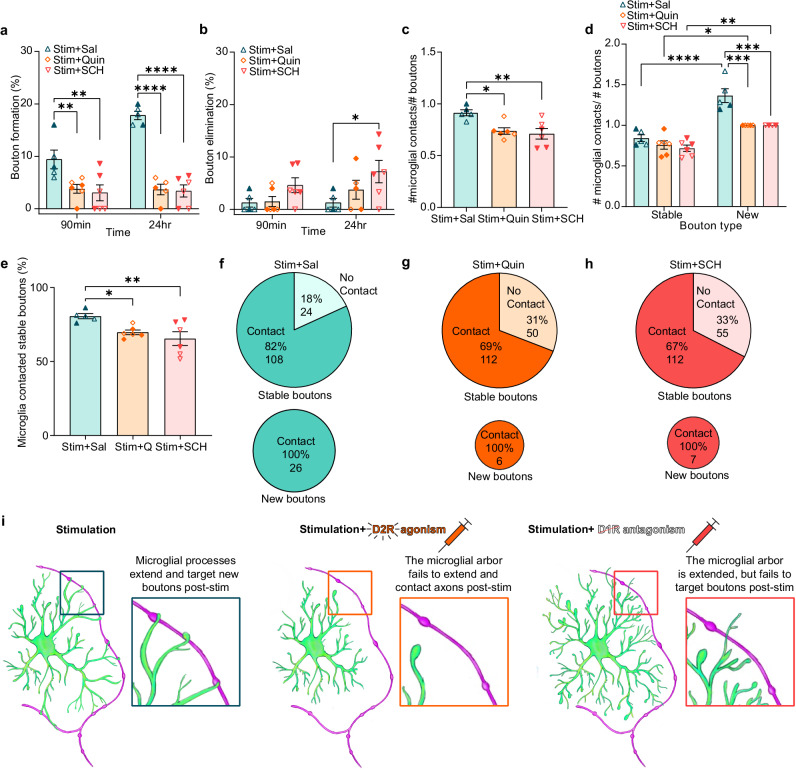
Manipulating DA receptor signaling blocks adolescent mesofrontal plasticity and reduces microglial contacts with DA boutons. **a** Both Quin and SCH blocked DA bouton formation post-phasic DA axon activation (n: Sal = 5, Quin = 6, SCH = 6, Mixed-effects model, Fixed effects [type III], Treatment *p* < 0.0001, F(2,14) = 30.59, Dunnett’s multiple comparisons 90 min: Stim + Sal v. Stim + Quin *p *= 0.0065, Stim + Sal v. Stim + SCH *p* = 0.0021, 24 hr: Stim + Sal v. Stim + Quin *p* < 0.0001, Stim + Sal v. Stim + SCH *p* < 0.0001). **b** SCH increased DA bouton elimination 24 hr post-phasic DA axon activation (n: Sal = 5, Quin = 6, SCH = 6 Mixed-effects model, Fixed effects [type III], Treatment *p* = 0.0650, F(2,14) = 3.343, Dunnett’s multiple comparisons, 24 hr Stim + Sal v. Stim + SCH  = 0.0144). **c** Both Quin and SCH reduced the number of microglial contacts per bouton (n: Sal = 5, Quin = 6, SCH = 6, One-way ANOVA *p* = 0.0069, F(2,14) = 7.264, Dunnett’s multiple comparisons test Stim + Sal v. Stim + Quin *p* = 0.0144 Stim + Sal v. Stim + SCH *p* = 0.0059). **d** Both Quin and SCH significantly reduced the number of contacts per new bouton compared to control mice (n: Sal = 5, Quin = 6, SCH = 6, Two-way ANOVA Bouton category x Treatment *p *= 0.0154, F(2,25) = 4.951, Šídák’s multiple comparisons test Stable Stim + Sal v. New Stim + Sal *p* < 0.0001 Stable Stim+Quin v. New Stim + Quin *p* = 0.0189, Stable Stim + SCH v. New Stim + SCH *p* = 0.0077, New Stim+Sal v. New Stim + Quin *p* = 0.0003, New Stim + Sal v. New Stim+SCH *p* = 0.0006). **e** Both Quin and SCH reduced the proportion of stable boutons that microglia contact (n: Sal = 5, Quin = 6, SCH = 6, one-way ANOVA, *p* = 0.0143, F(2,14) = 5.839, Holm-Šídák’s multiple comparisons test, Stim + Sal v. Stim + Quin *p* = 0.0306, Stim + Sal v. Stim + SCH *p* = 0.0096). **f**, **g**, **h** Pie charts showing pooled percentage (%) and number of stable and new boutons contacted by microglia in Sal (**f**), Quin (**g**), and SCH (**h**) conditions after opto-stimulation. Both Quin and SCH reduced the percentage of stable boutons contacted by microglia. New boutons were always contacted by microglia, but were less abundant after Quin and SCH treatment (illustrated by the size difference of the pie charts). **i** Summary diagram of how DR perturbations alter microglial dynamics and interactions with boutons. Multiple comparisons are two-sided. Graphs show mean ± S.E.M *p < 0.05, **p < 0.01, ***p < 0.005, ****p ≤ 0.0001. Individual points represent individual animals with females as hollow symbols and males as solid symbols. Source data are provided as a source data file.

Given the literature highlighting the relationship between microglial arborization and their ability to interact with synaptic elements^14–18^, we next tested if the disruption in microglial dynamics observed with pharmacological manipulation of D1R and D2R signaling in the CNS impacted how microglia interacted with DA boutons. We also investigated the potential concomitant changes in mesofrontal DA axon plasticity. We found that both D1R antagonism and D2R agonism blocked DA bouton formation after adolescent phasic DA axon stimulation (Fig. 5a, Mixed-effects model, Fixed effects [type III], Treatment *p* < 0.0001). At 24 hr post-stimulation, there was also an increase in bouton elimination in the SCH group, suggesting that D1R antagonism may have had a destabilizing effect on DA bouton maintenance (Fig. 5b Mixed-effects model, Fixed effects [type III], Treatment *p* = 0.0650).

While the Sal control group showed a similar degree of microglia contacts per bouton as in the previous no-treatment experiment (Fig. 3d), both Quin and SCH groups showed significantly lower contacts compared to the Sal group, suggesting a failure to recruit microglial processes to boutons (Fig. 5c, One-way ANOVA, *p* = 0.0069). While across all groups, microglia still contacted newly formed boutons more frequently than stable boutons, there was a significant reduction of this effect in both SCH and Quin groups (Fig. 5d, Two-way ANOVA Bouton category x Treatment *p* = 0.0154). There also appeared to be more microglial contacts with eliminated boutons than stable boutons, but the number of eliminated boutons observed was low (Supplementary Fig. 9). Furthermore, both Quin and SCH-treated microglia interacted with a smaller percentage of stable boutons than that in the Sal control (Fig. 5e one-way ANOVA, *p* = 0.0143). Across the groups, microglia contacted all new boutons, but again, there were far more new boutons in the Sal-treated controls compared to Quin and SCH groups (Fig. 5f–h).

These microglia bouton interaction findings were especially surprising in the SCH group, where microglial volume and surveillance were both higher than the Sal-stimulated control group. These findings suggest that increased microglial surveillance alone is not sufficient to produce increased microglia-DA bouton interactions. Our results indicate that microglial targeting to DA boutons is impaired when D1R receptor activity is blocked with SCH. Thus, our findings depict an interesting relationship between microglial dynamics and mesofrontal plasticity, whereby manipulating either D1R or D2R signaling alters microglial dynamics and blocks mesofrontal plasticity (Fig. 5i).

### Pharmacological inhibition of D2R in adults produces an adolescent-like microglial response to DA axon stimulation

**Fig. 6:**
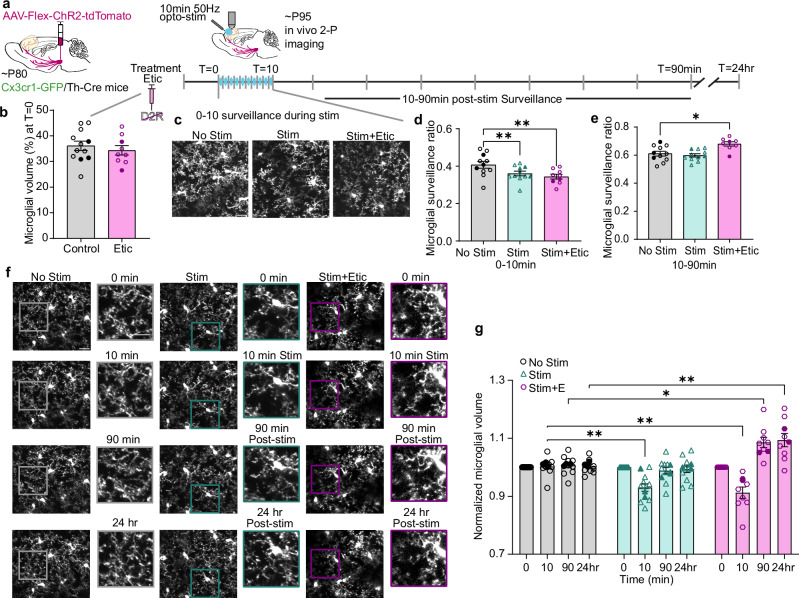
Inhibition of D2R in adulthood produces an adolescent-like microglial response to DA axon stimulation. **a** Timeline of dosing and imaging. **b** D2R antagonism (eticlopride 1 mg/kg, i.p. Etic) does not alter baseline parenchyma volume (n: Control = 12, Etic = 9 mice, two-sided unpaired *t*test t(19) = 0.6738, *p* = 0.5085). **c** Representative images of microglial surveillance during phasic DA axon stimulation. **d** Adult microglia respond to DA axon stimulation acutely, independent of D2R inhibition (n: No Stim = 11, Stim = 11, Stim + Etic = 9 mice, Mixed-effects model, Fixed effect [Type III] *p* = 0.003, F(1.827,16.44) = 14.53, Dunnett’s multiple comparisons test, No Stim v. Stim *p* = 0.0013, No Stim v. Stim + Etic *p* = 0.0059). **e** D2R inhibition during stimulation restores post-stimulation increase in microglial surveillance in adults (n: No Stim = 11, Stim = 11, Stim + Etic = 8 mice, Mixed-effects model, Fixed effect [Type III] *p* = 0.0029, F(3.126,16.28) = 8.696, Dunnett’s multiple comparisons test, No Stim v. Stim p = 0.7707, No Stim v. Stim + Etic *p* = 0.0211). **f** Representative images of microglial volume (Pop-outs highlight areas of interest). **g** D2R inhibition during stimulation restores the biphasic microglial response to mesofrontal activity (n: No Stim = 11, Stim = 11, Stim + Etic = 8 mice, Mixed-effects model, Fixed effect [Type III] Treatment x Time *p* < 0.0001, F(3.126,27.61) = 16.36, Dunnett’s multiple comparisons test, 10 min: No Stim v. Stim *p* = 0.0020, No Stim v. Stim + Etic *p* = 0.0022, 90 min: No Stim v. Stim+Etic *p* = 0.0163, 24 hr: No Stim v. Stim + Etic *p* = 0.0017). Scale bars 20 µm. Multiple comparisons are two-sided. Graphs show mean ± S.E.M *p ≤ 0.05, **p < 0.01, ***p < 0.005, ****p < 0.0001. No Stim, Stim and Stim + Etic experiments were conducted within the same animals. Individual points represent individual animals with females as hollow symbols and males as solid symbols. Source data are provided as a source data file.

After observing the robust microglial response to changes in DA signaling and mesofrontal plasticity during adolescence, we next tested if reinstating mesofrontal plasticity in adult animals would generate a similar microglial response. In adult mice, optogenetic stimulation of the VTA DA neurons alone does not generate mesofrontal DA plasticity, however, pairing stimulation with a D2R antagonist reinstates plasticity^9^. Based on this study, we treated adult animals (~ P95) with eticlopride (Etic,1 mg/kg, i.p.), a D2R antagonist, prior to our optogenetic stimulation paradigm (Fig. 6a). We found that Etic did not alter the baseline microglial volume in the parenchyma (Fig. 6b unpaired *t*test, *p* = 0.5085), suggesting that any baseline activation of D2Rs from tonic release of DA likely had little impact on microglial dynamics.

Next, we evaluated the response of adult microglia to mesofrontal DA axon stimulation. Like adolescent microglia, adult microglia acutely responded to phasic DA stimulation with a reduction in surveillance. This effect was independent of D2R inhibition (Fig. 6c, d, Mixed-effects model, Fixed effect [Type III] *p* = 0.0029, Supplemental Videos 12–14), suggesting that microglial retraction in response to DA stimulation may be mediated through other receptors or secondary signals. The impact of D2R inhibition became apparent in the post-stimulation period, as Etic restored the post-stimulation increase in microglial surveillance in adults (Fig. 6e, Fixed effect [Type III] p = 0.003, Supplemental Videos 15–17). Unlike the DA axon stimulation alone condition, stimulation paired with Etic generated the full biphasic microglial response that we had observed in our adolescent animals (Fig. 6f, g, Mixed-effects model, Fixed effect [Type III] Treatment x Time, *p* < 0.0001). Taken together, these results suggest that pharmacological inhibition of D2R in adults produces an adolescent-like microglial response to phasic DA axon stimulation.

### D2R inhibition in adults restores both mesofrontal axonal plasticity and microglial recruitment to DA boutons

**Fig. 7:**
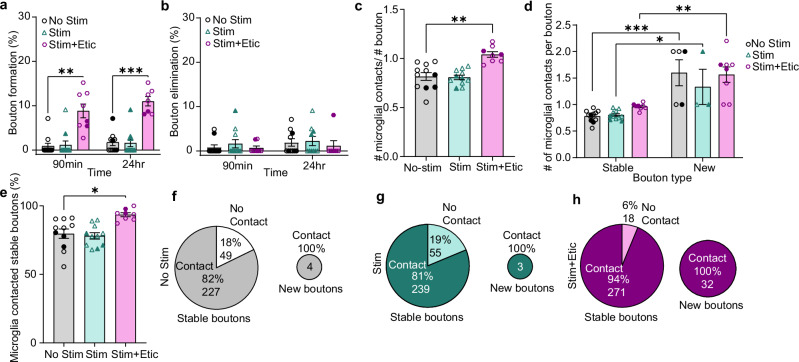
Inhibition of D2R in adulthood restores both mesofrontal axonal plasticity and microglial recruitment to DA boutons in adults. **a** D2R inhibition during DA axon stimulation reopens adult plasticity (n: No Stim = 11, Stim = 11, Stim + Etic = 8, Mixed-effects model, Fixed effects [type III], Treatment *p* < 0.0001, F(1.110,11.10) = 51.27, Dunnett’s multiple comparisons 90 min: No Stim v. Stim + Etic *p* = 0.0017, 24 hr: No Stim v. Stim + Etic *p* = 0.003). **b** Etic does not alter bouton elimination (n: No Stim = 11, Stim = 11, Stim + Etic = 8, Mixed-effects model, Fixed effects [type III], Treatment *p* = 0.6992, F(2,27) = 0.3626). **c** Etic increases the number of microglial contacts with boutons after DA axon stimulation (Mixed-effects model, Fixed effects [type III], *p* = 0.0002 F(1.516,12.89) = 19.92, Dunnett’s multiple comparisons No Stim v. Stim + Etic *p* = 0.0019). **d** Microglia preferentially make more contacts with new boutons than stable boutons (Mixed-effects model, Fixed effects [type III] bouton type *p *< 0.0001 F(1,13) = 51.59, Šídák’s multiple comparisons Stable v. New: No stim *p* = 0.0003, Stim *p* = 0.0338, Stim + Etic *p* = 0.0021). **e** Etic increases the proportion of stable boutons surveyed by microglia after DA axon stimulation (Mixed-effects model, Fixed effects [type III] *p* = 0.0042 F(1.518,12.90) = 9.812, Dunnett’s multiple comparisons No Stim v. Stim + Etic *p* = 0.0107). **f**–**h** Pie charts showing pooled percentage (%) and number of stable and new boutons contacted by microglia in No Stimulation (**f**), Stimulation (**g**), and Stimulation + Etic (**h**) conditions. Etic increased the percentage of stable boutons contacted by microglia. New boutons were always contacted by microglia, but were more abundant after Etic treatment (illustrated by the size difference of the pie charts). Multiple comparisons are two-sided. Graphs show mean ± S.E.M *p < 0.05, **p < 0.01, ***p < 0.005, ****p < 0.0001. No Stim, Stim and Stim + Etic experiments were conducted within the same animals. Individual points represent individual animals with females as hollow symbols and males as solid symbols. Source data are provided as a source data file.

Given the biphasic response in adult microglia with Etic administration, we next examined the effects of D2R inhibition on microglial interactions with DA boutons. First, we confirmed that as in our previous work, D2R inhibition during DA axon stimulation reinstates plasticity in adults, driving bouton formation without a concomitant change in elimination (Fig. 7a, b Formation Mixed-effects model, Fixed effects [type III], Treatment *p* < 0.0001; Elimination Mixed-effects model, Fixed effects [type III], Treatment *p* = 0.6992). We found that whereas DA axon stimulation alone did not promote microglial contacts with boutons, the addition of Etic increased the number of microglial contacts per bouton (Fig. 7c Mixed-effects model, Fixed effects [type III], *p* = 0.0002). This result implies that microglia are actively recruited when the circuit is being remodeled, rather than just in response to changes in neural activity.

As in our adolescent animals, microglia in adults preferentially made more contacts with new boutons compared to stable boutons, although few new boutons appeared in adults unless stimulation was paired with Etic (Fig. 7d Mixed-effects model, Fixed effects [type III] *p* < 0.0001). There were also more microglial contacts with eliminated boutons than stable boutons, but the number of eliminated boutons observed was low (Supplementary Fig. 10). Notably, Etic increased the proportion of stable boutons surveyed by microglia, again paralleling our stimulated adolescent animals (Fig. 7e Mixed-effects model, Fixed effects [type III] *p* = 0.0042). As in our adolescent cohorts, we found all new boutons were contacted by microglia, however, again, there were significantly more new boutons in the stimulated animals that received D2R inhibition than the unstimulated and stimulation alone groups (Fig. 7f–h). Thus, inhibiting D2R signaling in adult mice reinstates mesofrontal axonal plasticity with the recruitment of microglial processes to DA boutons. Taken together, our data from both adolescent and adult animals highlight the close relationship between microglial surveillance of axons and DA axon modification during mesofrontal plasticity.

### Pharmacological inhibition of microglial P2RY12 signaling prevents the biphasic microglial response to DA axon stimulation in adolescence

**Fig. 8:**
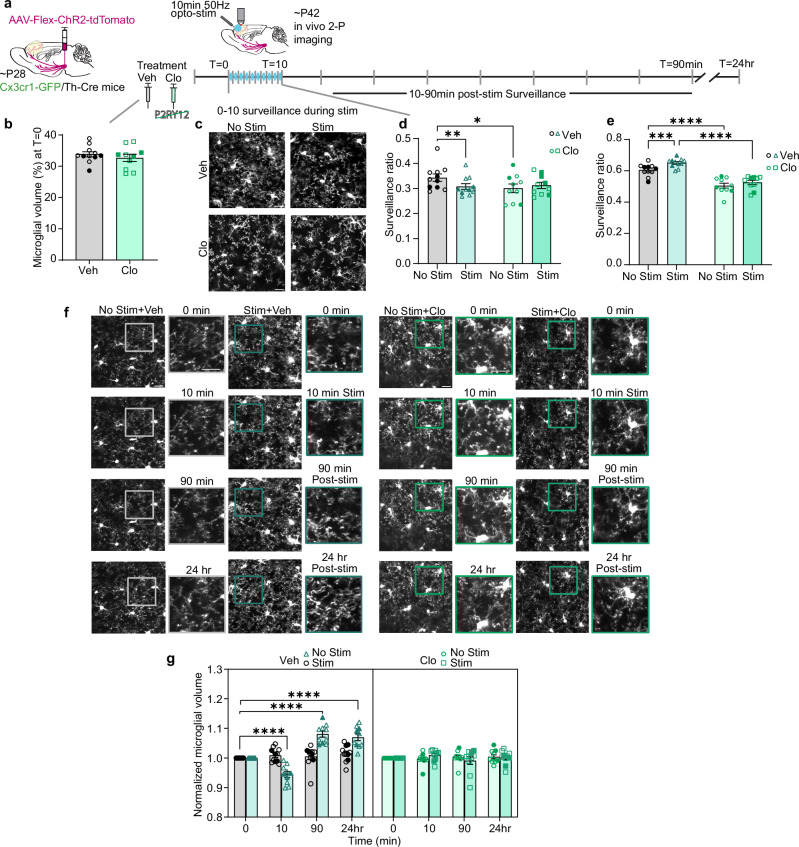
Pharmacological inhibition of microglial P2RY12 signaling prevents the biphasic microglial response to DA axon stimulation in adolescence. **a** Timeline of dosing and imaging. **b** P2RY12 inhibition (Clopidogrel 50 mg/kg, i.p, Clo.) does not alter baseline microglial volume as compared to vehicle (Veh) control (n: Veh = 11, Clo = 10 mice, two-sided unpaired *t* test t(19) = 0.8244, *p* = 0.4199). **c** Representative images of microglial surveillance during phasic DA axon stimulation. **d** Clo reduces microglial surveillance from 0–10 min during DA axon stimulation (n: Veh = 11, Clo = 10, two-way RM ANOVA *p* = 0.0069, F(1,19) = 9.184, Uncorrected Fisher’s LSD No Stim + Veh v. No Stim + Clo *p* = 0.0406 No Stim + Veh v. Stim + Veh *p* = 0.0038). **e** Clo prevents increased surveillance from 10–90 min post-DA axon stimulation (n: Veh = 11, Clo = 10, two-way RM ANOVA Main effect of treatment *p* < 0.0001, F(1,19) = 49.02, Uncorrected Fisher’s LSD No Stim + Veh v. No Stim + Clo *p* < 0.0001 No Stim + Veh v. Stim + Veh *p* = 0.0004 Stim + Veh v. Stim + Clo p < 0.0001). **f** Representative images of microglial volume. Rectangle pop-outs denote magnified areas. **g** Clo prevents the dynamic changes in microglial volume which accompany phasic DA axon stimulation (n: Veh = 11, Clo = 10 mice, Mixed-effects model, Fixed effects [type III], Time x Treatment x Stimulation *p* < 0.0001, F(3,48) = 26.27, Dunnett’s multiple comparisons test No Stim + Veh 0 min v. Stim + Veh 10 min *p *< 0.0001 No Stim+Veh 0 min v. Stim + Veh 90 min *p* < 0.0001 No Stim + Veh 0 min v. Stim + Veh 24 hr *p* < 0.0001). Scale bars 20 µm. Multiple comparisons are two-sided. Graphs show mean ± S.E.M *p < 0.05, **p < 0.01, ***p < 0.005, ****p < 0.0001. No Stim + Veh and Stim + Veh experiments were conducted within the same animals. No Stim + Clo and Stim + Clo experiments were conducted within the same animals. Individual points represent individual animals with females as hollow symbols and males as solid symbols. Source data are provided as a source data file.

After observing the tight coupling of microglial dynamics to axon bouton formation, we wanted to test if perturbing a microglial specific signaling system would inhibit the dynamic microglial response to DA axon stimulation (Fig. 8a). Previous work has highlighted the purinergic receptor P2RY12 as a central driver of microglial process chemotaxis toward sites of ATP release, responding not only to focal tissue injury but also to neuronal activity^24,38–40^. Of note, within the CNS, only microglia express P2RY12, making it an ideal target for microglia-specific manipulation^24^.

We first tested if P2RY12 inhibition would impact basal microglial physiology in M2. Previous work in V1 found that P2RY12 inhibition decreased microglial ramification^16^, however, no morphological change was observed in the hippocampus when P2RY12 was inhibited^38^. We found that clopidogrel (Clo), a selective P2RY12 antagonist (50 mg/kg i.p.), did not significantly alter baseline microglial volume when compared to vehicle control (Veh) (Fig. 8b Paired *t* test *p* = 0.4199). This suggests that P2RY12 inhibition in the adolescent frontal cortex does not have a significant impact on baseline microglial morphology. However, from 0–10 min Clo treatment significantly decreased microglial surveillance in the absence of optogenetic stimulation (Fig. 8c, d Two-way RM ANOVA *p* = 0.0069, Supplemental Video 18), suggesting an effect on baseline microglial dynamics. When compared to the Veh control, P2RY12 inhibition significantly lowers microglial surveillance, bringing both the Clo No Stim and Stim conditions down to the same level as the Veh Stim (Fig. 8d). Further, from 10–90 min this effect of Clo persists, with both unstimulated and stimulated Clo-treated microglia exhibiting significant decreases in parenchyma surveillance (Fig. 8e Two-way RM ANOVA *p* < 0.0001, Supplemental Video 19). This is interesting as previous work has not found a loss of microglial baseline surveillance with P2RY12 inhibition, however, of note, previous imaging was done in anesthetized preparations in other CNS regions^16,38^. It is possible that the decreased surveillance we see in the absence of stimulation with Clo administration is due to a basal loss of microglial process recruitment to ATP release from neuronal activity in the awake frontal cortex. In line with the changes in surveillance, Clo also eliminates the biphasic response of microglia to DA stimulation, mirroring the effects observed with DR signaling manipulation. Instead of dynamically adjusting, microglial volume remains stable throughout DA stimulation and the post-stimulation period when new boutons begin forming (Fig. 8f, g Mixed-effects model, Fixed effects [Type III] Time x Treatment x Stimulation *p* < 0.0001). Thus, pharmacological inhibition of P2RY12 is sufficient to completely eliminate the biphasic microglial response to adolescent mesofrontal DA axon stimulation.

### Microglial P2RY12 signaling is necessary for adolescent mesofrontal axon plasticity

**Fig. 9:**
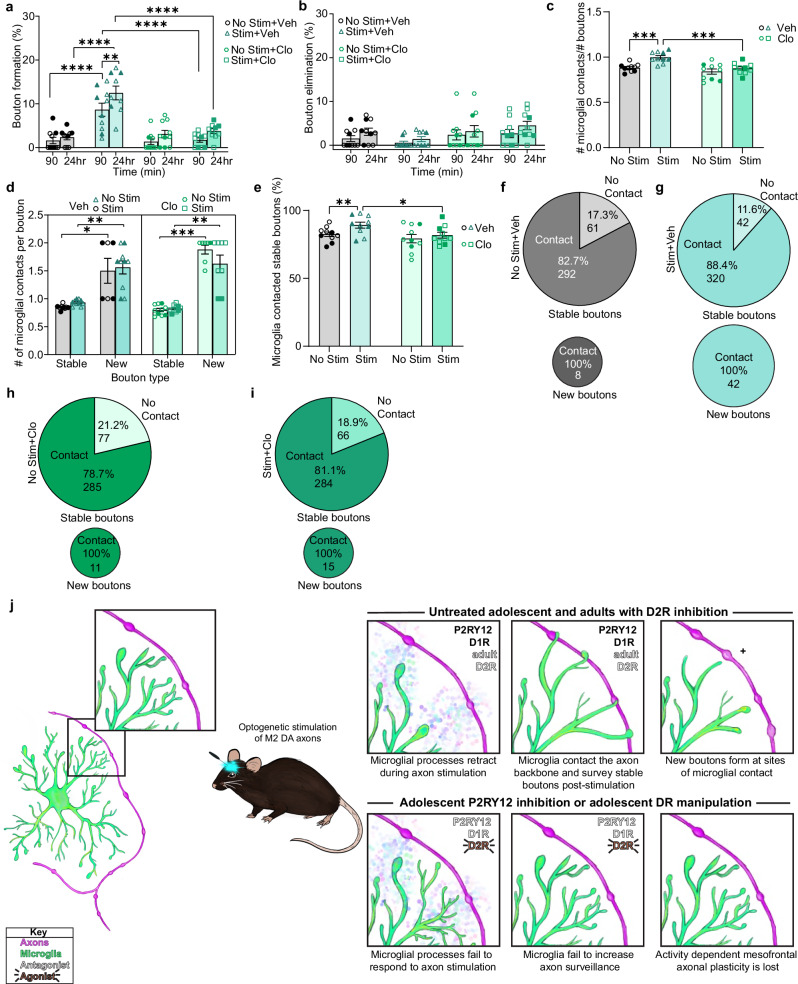
Microglial P2RY12 signaling is necessary for adolescent mesofrontal axon plasticity. **a** Clo blocked DA bouton formation post-phasic DA axon activation (n: Veh = 10, Clo = 10, Mixed-effects model, Fixed effects [type III], Treatment x Stim *p *< 0.0001, F(1,17) = 26.13, Šídák’s multiple comparisons No stim+Veh 90 min v. Stim + Veh 90 min *p* < 0.0001 No Stim+Veh 24 hr v. Stim + Veh 24 hr *p* < 0.0001 Stim + Veh 90 min v. Stim + Veh 24 hr *p *< 0.0001 *p* = 0.0032 Stim + Veh 90 min v. Stim+Clo 90 min *p* < 0.0001 Stim + Veh 24 hr v. Stim + Clo 24 hr *p* < 0.0001). **b** Clo does not alter bouton elimination (n: Veh = 10, Clo = 10, Mixed-effects model, Fixed effects [type III], Treatment x Stim *p* < 0.3105, F(1,18) = 1.093). **c** Clo reduces the number of microglial contacts per bouton (n: Veh = 10, Clo = 10, Two-way RM ANOVA p = 0.0275, F(1,18) = 5.753, Uncorrected Fisher’s LSD No Stim + Veh v. Stim + Veh *p* = 0.0001 Stim + Veh v. Stim + Clo *p* = 0.0002). **d** Newly formed boutons received more microglial contacts than stable boutons with Clo (n: Veh = 10, Clo = 10, Mixed-effects model, Fixed effects [type III] Main effect of Bouton Type *p* < 0.0001, F(1,18) = 119.9, Šídák’s multiple comparisons test Stable No Stim + Veh v. New No Stim + Veh  *p *= 0.0118 Stable Stim + Veh v. New Stim + Veh *p* = 0.0065 Stable No Stim + Clo v. New No Stim + Clo p = 0.003 Stable Stim + Clo v. New Stim + Clo p = 0.0017). **e** Clo reduces the proportion of microglial contacted stable boutons (n: Veh = 10, Clo = 10, Two-way RM ANOVA, Main effect of Stim *p* = 0.0072, F(1,18) = 9.176, Uncorrected Fisher’s LSD, No Stim+Veh v. Stim + Veh *p* = 0.0061 Stim + Veh v. Stim + Clo *p* = 0.0303). **f**–**i** Pie charts showing pooled percentage (%) and number of stable and new boutons contacted by microglia in No Stimulation + Veh (**f**), Stimulation + Veh (**g**), No Stimulation+Clo (**h**), and (**i**) Stimulation + Clo conditions. Clo reduced stable bouton contacts by microglia. New boutons were contacted by microglia, but Clo reduced the bouton formation rate to no stim levels (illustrated by pie chart size difference). **j** Summary diagram of microglial dynamics during mesofrontal plasticity. Multiple comparisons are two-sided. Graphs show mean ± S.E.M *p < 0.05, **p < 0.01, ***p < 0.005, ****p < 0.0001. No Stim Veh and Stim Veh experiments were conducted within the same animals. No Stim Clo and Stim Clo experiments were conducted within the same animals. Individual points represent individual animals with females as hollow symbols and males as solid symbols. Source data are provided as a source data file.

With the observed perturbations of the microglial response to DA axon stimulation, we wanted to know if the loss of the dynamic microglial response would inhibit mesofrontal plasticity. Indeed, with Clo inhibition of P2RY12, DA bouton formation was impaired post-stim with no concomitant change in elimination (Fig. 9a, b Mixed-effects model, Fixed effects [Type III] p < 0.0001; Mixed-effects model, Fixed effects [Type III] p = 0.3105). Clo treatment maintains the bouton contact frequency at the level observed in unstimulated control animals, despite optogenetic stimulation (Fig. 9c, Two-way RM ANOVA *p* = 0.0275). Clo did not abolish the preferential contact of microglia with new boutons, but there were few new boutons in Clo-treated mice compared to controls (Fig. 9d Mixed-effects model, Fixed effects [Type III] Main effect of Bouton Type *p* < 0.0001). Clo treatment also did not alter the microglial preference for more frequently contacting eliminated than stable boutons, though there were few eliminated boutons (Supplementary Fig. 11). Furthermore, Clo treatment prevented the increased surveillance of the stable pool of boutons post-stimulation (Fig. 9e, Two-way RM ANOVA Main effect of stimulation p = 0.0072). Thus, inhibition of P2RY12 signaling does not completely abolish microglia bouton contact, but rather specifically prevents the activity-dependent recruitment of microglial processes to DA boutons (Fig. 9f–i). Notably, pharmacological inhibition of P2RY12 not only altered microglial dynamics but also blocked mesofrontal DA axon plasticity. As microglia are the only CNS cells that express P2RY12^24^, this suggests that intact microglial purinergic signaling is necessary for mesofrontal DA plasticity. Taken together, our findings show that microglia are highly sensitive to adolescent DA signaling and that both intact DR signaling and microglial P2RY12 signaling are required for activity-dependent DA axon plasticity in the frontal cortex (Fig. 9j).

## Discussion

In this study, we demonstrate that frontal cortical microglia are sensitive to DA signaling and that microglial surveillance is required for adolescent mesofrontal plasticity. Our study integrated a range of advanced technical approaches, including dual genetic labeling of microglia and DA projections, longitudinal in vivo two-photon imaging of glial-neural interactions in awake animals, ethologically relevant behavioral paradigms, projection-specific optogenetic manipulation, and complementary pharmacological interventions. We found that adolescent frontal cortical microglia exhibit a unique biphasic response to DA axon stimulation with an initial retraction and a subsequent extension of their processes. This robust process extension facilitated increased microglial surveillance of DA boutons. Notably, microglial contacts along the axon backbone preceded new bouton formation. Perturbation of either D1 or D2 signaling in adolescence was sufficient to impair both the biphasic microglial response and mesofrontal plasticity. Moreover, inhibiting D2 signaling in adulthood produced an adolescent-like microglial response to phasic DA stimulation and reinstated mesofrontal plasticity. Finally, blocking the microglia receptor P2RY12 eliminated the biphasic microglial response to DA axon stimulation and prevented mesofrontal axon plasticity. Our findings highlight a bidirectional interaction between microglial surveillance and activity-dependent DA bouton formation, suggesting that microglia not only respond to DA signaling but also actively promote adolescent mesofrontal plasticity.

### Microglial surveillance and DA bouton formation in the frontal cortex

The advent of in vivo two-photon microscopy techniques enabled new insights into the non-pathological dynamics of microglial surveillance^41,42^ and the interactions of dynamic microglial processes with neuronal elements in the intact CNS^43,44^. Since the initial discovery of microglial surveillance of neural elements, extensive work has shown that microglia are essential participants in developmental plasticity^14,45,46^. Much of the previous in vivo and ex vivo work has identified microglia as facilitators of dendritic spine or synaptic bouton removal during periods of circuit development or remodeling^44,47,48^. As immune cells, microglia are naturally suited to removing CNS elements. Microglial immune machinery, such as the complement system, is frequently repurposed for synaptic pruning during health and disease^14,49^. In the context of the existing literature, the present study provides several insights into microglial-neuron dynamics. First, we present an in vivo description of microglial interactions with neuromodulatory boutons, which are not associated with well-defined postsynaptic structures like glutamatergic or GABAergic boutons^11^. Second, we show that microglial contact with axonal boutons is associated with bouton formation rather than removal^44^.

We identified many instances of tight temporal coupling between microglial contact and bouton formation following axon stimulation, suggesting that rapid recruitment of microglial processes to the axon backbone may be a critical step in initiating bouton formation. Although microglia make more frequent contacts with both newly formed and eliminated boutons compared to stable ones, eliminated boutons remain rare—even when combining multiple datasets. Importantly, microglial process volume correlates with bouton formation rates but not with elimination rates (Supplementary Fig. 12), indicating that microglial surveillance primarily supports frontal DA bouton formation during adolescence. The consistent observation that bouton formation is preceded by microglial contact with the axon backbone suggests that microglial processes may remodel the extracellular matrix (ECM)^50^ or trigger axonal reorganization to facilitate new bouton formation. Moreover, the requirement of microglial P2RY12 signaling for activity-dependent bouton formation—without affecting bouton elimination—further implicates microglia as essential drivers of mesofrontal plasticity.

Based on previous studies, multiple candidate molecular mechanisms may underlie how microglia facilitate bouton formation. Microglia possess a wide repertoire of receptors and signaling systems that allow them to guide neural plasticity and development^47^. In the hippocampus, neuronal interleukin-33 signaling upregulates ECM proteases in microglia, promoting ECM remodeling necessary for dendritic spine formation^50^. In the developing somatosensory cortex, microglial contacts with dendrites have been associated with calcium transients and actin accumulation preceding filipodia formation^51^. In the motor cortex, microglia facilitate dendritic spine formation during motor learning through the release of brain-derived neurotrophic factor^17^. These examples suggest that the mechanisms by which microglia shape synaptic remodeling and plasticity may not be uniform across CNS regions^47^. Although this mechanistic diversity complicates our understanding of microglia-neuron interactions, it also presents opportunities for highly specific, regionally targeted manipulations. In our study, wheel running evoked a regionally selective microglial response in the frontal cortex, demonstrating the potential to engage frontal cortical microglia through naturalistic rewarding behavior. While P2RY12 signaling is essential for recruiting microglial processes to stimulated DA axons, additional molecular pathways linking microglial surveillance to DA bouton formation remain promising areas for future research.

### Responses of frontal cortical microglia to DA signaling in adolescence

As immune cells acutely sensitive to changes in the CNS milieu, microglia are well-positioned to mediate rapid and targeted modifications in neural circuitry. However, fundamental questions remain—not only about how microglia interact with neuronal elements, but also about the molecular signals that govern their involvement during sensitive periods of development. Notably, prior studies have not provided in vivo evidence of whether or how microglia respond to DA signaling. Our study provides multiple lines of in vivo evidence indicating that frontal cortical microglia respond to DA signaling during adolescence. First, using voluntary wheel running as a naturally rewarding behavioral paradigm for mice, we found that this experience promotes microglial process outgrowth specifically in the adolescent frontal cortex, which receives extensive DA innervation, but not in the adolescent visual cortex, which receives little DA innervation. Second, by directly stimulating frontal DA axons with a phasic activity pattern typically associated with reward, we discovered a biphasic microglia response characterized by transient arbor retraction during stimulation, followed by sustained arbor extension post-stimulation. Third, pharmacological manipulation revealed that D1 receptor antagonism maintains microglia arbors in an extended state, whereas D2 receptor agonism maintains microglia arbors in a retracted state—both preventing the biphasic microglial response to frontal DA input. While previous studies have shown that microglia surveillance of the parenchyma can be inhibited by noradrenergic signaling^15,21^, this biphasic microglial response has not been reported before. The sustained increase of microglial surveillance after rewarding experience or DA axon stimulation represents an additional finding of this study.

DA may directly influence microglial physiology, as previous studies have shown that microglia express both D1- and D2-type DA receptors (DRs)^25,27,37,52^, and that DR signaling affects microglial membrane conductance, chemotaxis, phagocytosis, and interleukin signaling in vitro^25,53,54^. Only a subset of cultured microglia responded to DA, suggesting potential cellular heterogeneity^25^. Recent large-scale spatial transcriptomic studies further confirm *Drd1* and *Drd2* expression in subsets of frontal cortical microglia^27,37^. Complementing these findings, previous work has highlighted the role of the K^+^ channel THIK-1 in maintaining microglial resting membrane potential and basal surveillance of the CNS^38^. Moreover, in vitro studies have shown that DR signaling alters microglial membrane properties via inward-rectifying K_ir_ conductance^25^. Together, these findings raise the possibility that microglial DRs may interact with THIK-1 or K_ir_ channels to modulate microglial surveillance of the parenchyma.

It is also possible that DA may influence microglial dynamics indirectly through other cell types and molecular pathways. Both excitatory and inhibitory neurons in the frontal cortex express DA receptors and respond to DA signaling^5,36^. Microglia are sensitive to changes in fast excitatory and inhibitory neurotransmission, as well as to activity-dependent local ATP release^47^. In support of this, recent work has found that microglial protrusions are recruited to thalamocortical synapses in an activity-dependent manner by purinergic signaling through the microglial P2RY12 receptor^40^. Extending these findings, our study shows that frontal cortical microglia exhibit a biphasic response to DA signaling, characterized by an initial process retraction followed by sustained extension. D1 inhibition leads to extended microglial processes at baseline and prevents the biphasic response to subsequent DA stimulation. In addition, microglial recruitment to DA boutons is disrupted despite the presence of extended processes. These observations suggest that D1 signaling may mediate initial process retraction and prime microglia for subsequent re-targeting, while activity-dependent co-release of ATP and purinergic signaling may guide chemotaxis towards activated DA axons^40,55^. Importantly, inhibition of P2RY12 signaling reduced microglial surveillance and abolished axonal bouton plasticity, indicating that intact DR and P2RY12 signaling are both necessary for the biphasic microglial response to DA stimulation and for axonal remodeling. While P2RY12 expression is specific to microglia within the CNS^24^, the cellular sources of DR signaling are more complex. In addition to neurons, astrocytes also express DRs^56^ and may contribute to microglial responses to mesofrontal DA signals^57^. An important direction for future research will be elucidating how DR signaling from neurons, astrocytes, and microglia converges and ultimately regulates microglial responses to DA.

### Age-dependent mesofrontal plasticity and microglial DA responses

Adolescence is increasingly recognized as a sensitive period for frontal cortical development^1^. Microglia have been implicated as key regulators of such sensitive periods across multiple cortical areas, including the frontal cortex^16,58^. In this study, we found that D2 receptor signaling plays a critical role in regulating age-dependent microglial function and axonal plasticity. Specifically, D2 agonism in adolescence suppresses microglial surveillance and bouton formation, while D2 antagonism in adults reinstates an adolescent-like microglial surveillance state and promotes mesofrontal plasticity. Notably, D2 inhibition in adults does not alter the basal state of microglial processes or the initial retraction during DA stimulation, but selectively restores the post-stimulation process extension and bouton contact that are otherwise restricted to adolescence. These findings suggest that age-dependent changes in D2 signaling^5^ may limit microglial surveillance and activity-dependent plasticity in the adult frontal cortex. As discussed above, the cellular sources of D2 signaling are complex, whereas P2RY12 is expressed specifically in microglia within the CNS^24^ and is required for microglial surveillance of DA boutons. How D2R and P2RY12 signaling interact to regulate age-dependent microglial DA responses and mesofrontal plasticity remains an intriguing question for future research. Additionally, although we did not directly assess the activity of newly formed boutons in this study, our previous work has shown that mesofrontal structural plasticity enhances circuit activity^9,13^. Future research may further explore the potential to enhance mesofrontal plasticity in adulthood as a strategy for functional restoration following disease or injury.

In summary, this work identifies DA signaling as a critical regulator of microglial surveillance and adolescent plasticity in the frontal cortex. Moreover, intact microglial purinergic signaling is essential for mesofrontal DA circuit remodeling. Together, these findings establish a foundation for future studies investigating how activity-dependent microglial interactions with DA circuits influence brain development and function in both health and disease.

## Methods

### Animals

All experimental protocols were approved by the Institutional Animal Care and Use Committee at the University of Rochester (Protocol 2018-035) and followed the National Institutes of Health guidelines. All experiments were conducted on both male and female mice on a C57BL/6 background between P28-P52 (adolescent) and P70-P100 (adult). CX3CR1^GFP^ (JAX 005582)^59^ were crossed to the TH-Cre^60^ line to allow for both microglial visualization and specific viral targeting for labeling and manipulation of dopaminergic axons. Mice were housed in a temperature-controlled room (64–79 °F) with 30–70% relative humidity, maintained on a standard 12 h light/12 h dark cycle, and provided with standard chow and water ad libitum. In all figures, male mice are solid symbols and females are hollow, the numbers of each are also included in the figure legends.

### Pharmacologic agents

Quinpirole, a D2 receptor agonist (Sigma, 1 mg/kg), SCH23390, a D1 receptor antagonist (EMD Millipore,1 mg/kg), and eticlopride, a D2 receptor antagonist (1 mg/kg), were used to acutely alter dopaminergic receptor signaling during in vivo optogenetic imaging experiments. Solutions were prepared fresh on the day of imaging using sterile saline. Each of these agents were injected i.p. ~ 15 min prior to phasic optogenetic stimulation in their respective experiments. Clopidogrel, a P2RY12 antagonist (Selleck, 50 mg/kg), was administered 2 h prior to imaging.

### Cranial window surgery and viral targeting of VTA dopaminergic neurons

Mice were kept under isoflurane anesthesia (2-3%) for the duration of the surgical procedures, and a heating pad was used to maintain body temperature at 37 °C during surgery. Mice were mounted and the head fixed in a stereotaxic frame for all surgical procedures. All surgical procedures adhered to aseptic technique. After a scalp incision and clearing of the connective tissue, a 0.5 mm drill bit (FST) was used to drill a small hole through the skull for injection into the VTA. Either 375 nl AAV9-CAG-FLEX-tdTomato (wheel running and optogenetic control, Addgene 28306-AAV9, 3.8 × 1013 gc/mL) or AAV9-CAG-Flex-ChR2-tdTomato (optogenetic stimulation, Boston Children’s Hospital Viral Core, 4.82 × 1013 gc/mL) was injected through a glass micropipette connected to a micropump (from bregma: AP − 3.2, ML 0.5, and DV 4.5 mm, for both adolescent and adult experiments). After the VTA injection in the same surgical session, a 3 mm craniotomy was opened over M2 using a dental drill. As previously described^15^, a 5 mm coverslip attached to a 3 mm coverslip (Electron microscopy sciences) by UV glue (Norland Optical Adhesive, Norland) was fixed into the craniotomy using dental cement (C&B Metabond, Parkell). Dental cement was then used to cover the remaining surface of the skull, seal the incision site, and affix a small metal head plate. Animals were given slow-release buprenex (5 mg/kg subcutaneously every 72 h) and monitored daily during the 72 h postoperative period. Animals were imaged no sooner than 2 weeks post-op to allow for full surgical recovery and viral construct expression. After the completion of all imaging experiments, all animals were perfused to verify viral labeling of the VTA. Any animals lacking viral labeling of the VTA were excluded from further analysis.

### Two-photon microscopy

A two-photon microscope (FV1000, Olympus) was used for all in vivo imaging (excitation laser: 900 nm). All imaging was collected with a x25 water immersion lens (NA1.05) in head-fixed awake animals with a 380–560 filter for GFP-labeled microglia and a 575–630 filter for tdTomato-labeled dopaminergic axons. Mice received three consecutive days of head restraint training prior to in vivo imaging sessions, progressing from 30 min to 90 min over the course of the training sessions. A 1x image was taken at the first time point for each animal in the experiment to facilitate locating the same ROI again at subsequent time points. For microglial occupancy and dopaminergic bouton analysis in wheel running experiments, individual z stacks of the same ROI were taken at 3x digital zoom with a 1 µm z step at each time point. For optogenetic experiments, repeated z-stack imaging was done at an interval of every minute for 10 min (during stimulation) and every 10 min for 90 min (post-stimulation). Further details on the separate imaging parameters for these experiments are detailed below. All image analysis was done offline using ImageJ, blind to condition as described in Stowell et al.^15^ and as described below.

#### Wheel running

An initial baseline z-stack ROI (100–150 µm, 1 µm z step) containing both labeled axons and microglia was collected, and then the animal was returned to its home cage, or placed on a running wheel (Med Associates) for 2 h^9^. Med Associates Software was used to automatically record the number of revolutions the animal ran on the wheel. Any animals that ran fewer than 250 revolutions were excluded from further analysis. Immediately after the 2 h period, the ROI was imaged again, and then it was imaged a final time at 24 h. Images from the wheel running experiment were analyzed offline using ImageJ for bouton dynamics and microglial volume, as described in the respective sections below.

#### Optogenetic stimulation

For stimulation experiments, two initial ROIs were located and imaged at baseline prior to stimulation. The ROI1 z-stack was used to observe the acute microglial response to DA axon stimulation between stimulation pulses (20 µm, 1 µm z step) and the ROI2 stack was used for the microglial bouton interaction measurements (60–80 µm, 1 µm z step. For optogenetic stimulation, an optical fiber (200 µm diameter, Thor Laboratories) was connected to a 473 nm solid-state laser diode (CrystaLaser) with ~ 20 mW output from the fiber. This optical fiber was then positioned directly over the M2 cranial window glass coverslip to deliver phasic stimulation (50 Hz pulse train, 3 ms/pulse, 10 pulses/train, 1 train/min for 10 min). To measure the acute microglial response to DA axon stimulation, microglia in ROI1 were imaged every minute between the pulses for the 10 min stimulation period (10 stimulation z-stacks). To image microglial interactions with boutons and bouton dynamics, after the cessation of stimulation, ROI2 was imaged at 10 min intervals for 90 min post-stimulation (9 post-stimulation z-stacks). ROI2 was also imaged again at 24 h for a final time point. Images from the optogenetic experiments were analyzed offline using ImageJ for bouton dynamics, microglial contacts with boutons, microglial volume, and microglial surveillance as described in the respective sections below.

#### Calcium imaging

To verify that the optogenetic stimulation paradigm was effectively stimulating the mesofrontal circuit, we used in vivo two-photon calcium imaging to capture the cortical response to our stimulation paradigm. We used Th-cre mice and injected 375 nl AAV2/9-CAG-Flex-ChR2-tdTomato into the VTA and infused 450 nl AAV9.CamKII.GCaMP6s.WPRE.SV40 (Addgene 107790-AAV9, 2.5 × 10^13 GC/mL) into M2 (Bregma, AP 1.7, ML 0.8) from the pial surface using a glass micropipette connected to a micro-syringe pump as previously described^61^. A cranial window and head plate were affixed to the skull as described in the above surgical procedures. Optogenetic stimulation was done following the same phasic parameters. During stimulation, a time series of images (~ 40 s, 0.351 s/frame) were collected between each stimulus train for a total of 10 time series between the pulses. Analysis was conducted offline using custom MATLAB scripts. Briefly, baseline fluorescence (F0) was defined as the average fluorescent signal (Ft) in the first 15 s of the time series. Changes in the calcium signaling (ΔF/F) was calculated as (Ft-F0)/F0. We compared the average of the 10 time series to the baseline calcium activity in the ROI.

#### Microglia sholl analysis

To analyze microglial morphology in vivo, 3 microglia were selected per animal in the time 0 image. The same microglia were found and analyzed in the 2 h and 24 h time points after wheel running or home cage control. For each individual microglia selected, an individual z-projection was created in ImageJ while referencing the original imaging stack to ensure the entire arbor was captured. Microglial processes were then manually traced to ensure the entirety of the arbor was present in the thresholded image subjected to Sholl analysis (ImageJ, Sholl plugin). The average of the 3 Sholl profiles was calculated for each animal at each time point, and these values represented that animal in group analysis. For statistical analysis, we calculated the area under the curve (AUC) and the maximum intersection number as in previous work^15^.

#### Microglial volume (%) and surveillance

All analysis was done blind to condition. For all microglial analysis, the z stacks collected during each imaging session were processed in ImageJ to register any drifts over time and remove movement artifact from animals moving during imaging (Correct 3-D drift, ImageJ). Time course images were also corrected for intensity shifts associated with photo bleaching (Bleach correct, ImageJ). The same section of the z-stack was maximum intensity projected at each time point in an imaging series (20–30 µm thick), and lateral motion artifacts were corrected (Stackreg plugin, ImageJ). To quantify microglial volume at each time point in an image series, the z projections were binarized (Threshold, ImageJ), and the percent of microglia pixels out of the total ROI pixels was found for each time point. All volume values were then normalized to the microglial volume at the *t* = 0 image. Surveillance analysis was performed as previously described^15,16^. Briefly, for surveillance analysis, consecutive z projections from each time point were collapsed through the maximum-intensity z-projection function in ImageJ. This image was binarized, and surveillance was measured as the ratio of microglia pixels out of the total pixels in the image. Surveillance is a composite readout which captures changes in both motility and morphology. The complexity of the microglial arbor and the movement of individual processes contribute to how effectively microglia sample the parenchyma^15^.

#### Bouton formation and elimination

To quantify bouton dynamics in all time-lapse in vivo two-photon images, boutons at each time point were labeled with the multi-point tool in ImageJ. Boutons were identified as bright swellings along axons larger than 0.5 µm^2^ in size with a fluorescent intensity 2.5-fold brighter than the adjacent axon backbone, as in previous work^9^. Bouton identification in each image was done blind to the condition. The percent bouton formation was calculated as the number of boutons present at time point 2 (either 90 min, 2 hr, or 24 hr), but not time point 1 (0 min), divided by the total number of boutons present at time point 1. The percentage of eliminated boutons was calculated as the number of boutons present at time point 1 but not at time point 2, divided by the total number of boutons present at time point 1.

#### Microglial bouton contact analysis

All contact analysis was performed blind to condition. To visualize microglial contacts with dopaminergic axons, the GFP (microglia channel) was assigned the color green, and the tdTomato (axon channel) was assigned the color magenta. Microglial contacts with boutons or the axon backbone were determined by manually stepping through the z-stack (60–80 µm, 1–µm z step) of the merged channels. A contact was counted as the colocalization of microglial and axonal pixels. For each animal, 10–50 boutons were evaluated for microglial contact per imaging condition collected (only axons visible at all time points were evaluated). While viral labeling efficacy varied between animals, within the same animal, similar numbers of boutons were counted in each condition. Animals with a higher number of labeled boutons had a greater number of boutons analyzed for contact in both unstimulated and stimulated conditions, and vice versa for less robustly labeled animals. The multi-point tool in ImageJ was used to label and number boutons for time course tracking. All identifiable boutons were tracked through each time point to establish if it was stable, newly formed or eliminated. The total number of microglial contacts, whether the contact preceded a bouton change, and the interval from contact to bouton formation, were recorded. The number of contacts per bouton was calculated to determine the frequency of microglial bouton contacts. The proportion of boutons contacted was used to assess what percentage of the total boutons in a given ROI were contacted by microglia. The contact frequency and proportion of contacted data were subdivided by the categories of stable bouton, newly formed bouton, or eliminated bouton. For newly formed boutons, contacts with the axon backbone at the site of the new bouton prior to bouton formation were logged as bouton contacts and counted in the total count. To avoid issues of photo bleaching from repeated imaging, unique ROIs were selected for repeated measures within the same experimental animal.

### Histology

For histological experiments, mice were euthanized with an overdose of sodium pentobarbital (Euthasol, Virbac). Intact brains were collected after transcardial perfusion with PBS and fixed overnight in paraformaldehyde (4%). Coronal sections of tissue were cut on a vibratome at 70 µm thickness. Sections were then mounted and cover-slipped. For confirmation of viral injection efficacy, the VTA was viewed at 10x and 25x magnification using a confocal microscope (Olympus FV 1000). We also assessed the utility of the CX3CR1^GFP^/DAT-Cre (JAX: 006660)^62^ /Ai14 (JAX:007914)^63^ line for our imaging purposes. For this line, both the VTA and frontal cortex were examined and compared to the labeling seen in our virally injected CX3CR1^GFP^/Th-Cre mice. Sample images of both our injected CX3CR1^GFP^/Th^Cre^ and CX3CR1^GFP^/DAT-Cre/Ai14 mice were collected as z stacks with a step size of 1 µm using the confocal microscope. Due to the profound absence of mesofrontal dopaminergic axon labeling in our CX3CR1^GFP^/DAT-Cre/Ai14mice and the ectopic labeling of cortical neurons, no formal quantification was done using this line.

### Statistics

Statistical analyses were performed in Prism 10 (GraphPad, La Jolla, CA). All n-values represent individual animals, and our sample sizes were based on the norms for similar experiments found in the literature^13,15,16^. All individual values are represented on our plots with the mean ± s.e.m. included. For all analyses, α = 0.05. Statistical differences between two means were calculated using two-tailed unpaired or paired *t* tests. Statistical differences between multiple means were calculated using one-way or two-way ANOVAs with Holm-Šídák, Šídák, Fisher's LSD or Dunnett’s post hoc comparisons where appropriate. In cohorts with multiple time points, with occasional missing time points, Mixed-effects model (REML) Fixed effects (type III) was used in place of ANOVA to allow for missing values.

### Reporting summary

Further information on research design is available in the Nature Portfolio Reporting Summary linked to this article.

## Supplementary information

Supplementary Information

Description of Additional Supplementary Files

Supplemental Video 1

Supplemental Video 2

Supplemental Video 3

Supplemental Video 4

Supplemental Video 5

Supplemental Video 6

Supplemental Video 7

Supplemental Video 8

Supplemental Video 9

Supplemental Video 10

Supplemental Video 11

Supplemental Video 12

Supplemental Video 13

Supplemental Video 14

Supplemental Video 15

Supplemental Video 16

Supplemental Video 17

Supplemental Video 18

Supplemental Video 19

Reporting Summary

Transparent Peer Review file

## Source data

Source Data

## Data Availability

All data are available from the corresponding authors on request. Source data are provided in this paper.

## Code availability

Matlab scripts used for calcium imaging analysis are available at: https://github.com/RDStowell/WangLab-MatLabScripts.
