# Supplementary material for: Dopaminergic signaling regulates microglial surveillance and adolescent plasticity in the mouse frontal cortex
Source: Nat Commun. 2025 Aug 26;16:7974. doi: 10.1038/s41467-025-63314-4 (PMC12381247; doi:10.1038/s41467-025-63314-4)
Supplement: Supplementary file 1 — Supplementary Information [file 41467_2025_63314_MOESM1_ESM.pdf]

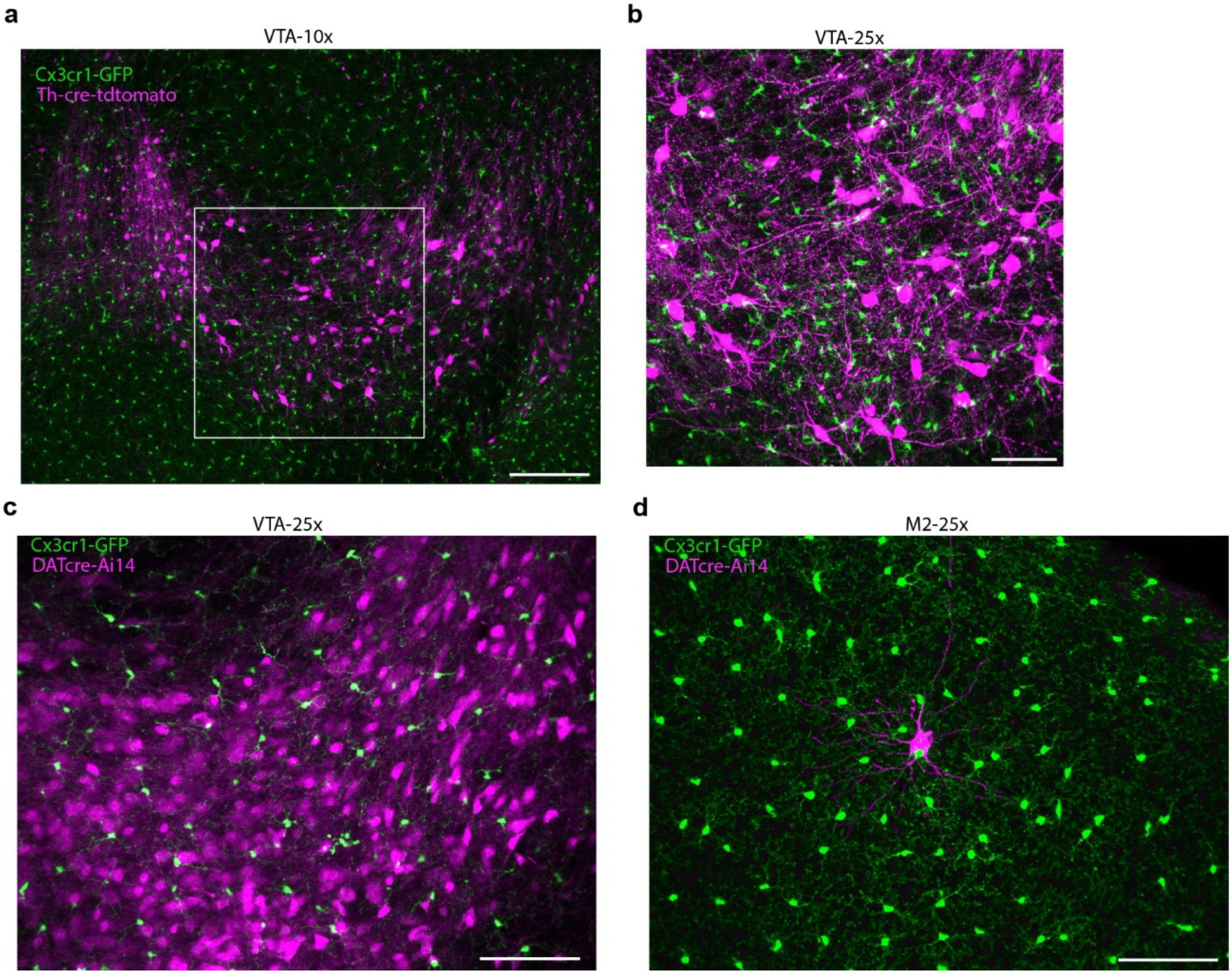

Supplemental Figure 1| Cx3cr1^{GFP}/Th-Cre mice are more effective for imaging the mesofrontal DA circuit than Cx3cr1^{GFP}/DAT-Cre/Ai14 mice. (**a**) Representative maximum intensity Z-projection of a 10x confocal image of VTA in Cx3cr1^{GFP}/Th-Cre mice with AAV-CAG-FLEX-tdTomato injection into the VTA (Th+ neurons-magenta, microglia-green) (**b**) Maximum intensity Z-projection of a 25x image of the square ROI from panel a. (**c**) Representative image of the VTA from a Cx3cr1^{GFP}/DAT-Cre/Ai14 mouse (DAT+ neurons-magenta, microglia-green). (**d**) Representative image of M2 frontal cortex in a Cx3cr1^{GFP}/DAT-Cre/Ai14 mouse. Note the lack of axonal labeling and the presence of an ectopically labelled cortical neuron. (DAT+ neuron-magenta, microglia-green). Scale bars 100μm (a,c,d) and 80μm (b).

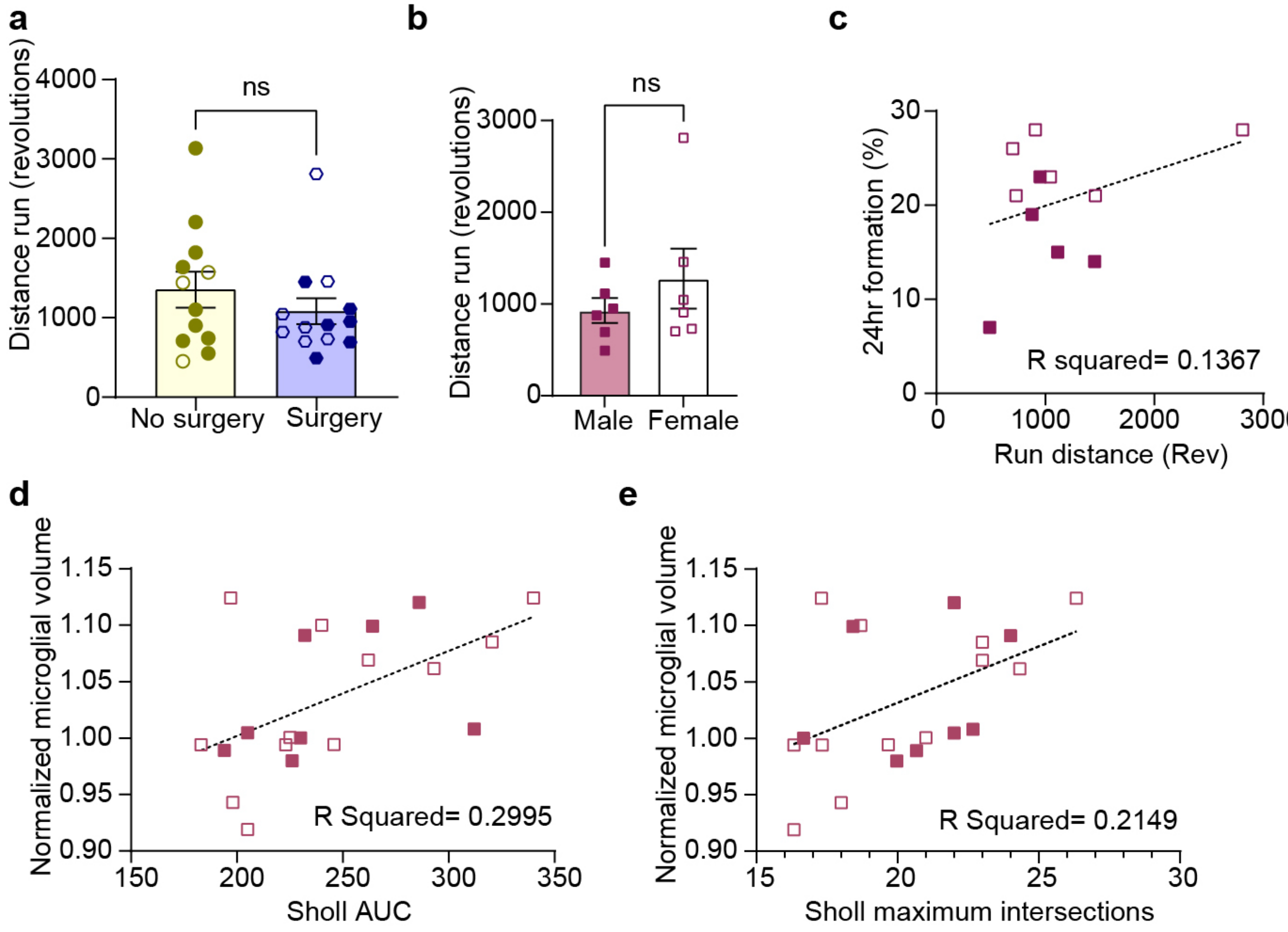

Supplemental Figure 2| Chronic cranial window preparations do not alter wheel running behavior and the normalized microglial volume correlates with Sholl analysis results. (**a**) Chronic cranial window implantation does not significantly alter wheel running (n: No surgery=12, surgery=13, two-tailed unpaired t-test, p=0.3306, t(23)=0.9939). (**b**) Females do not run significantly more than males (n: male=6, female=6; two-tailed unpaired t-test, p=0.3513, t(10)=0.9777). (**c**) There appears to be a positive correlation between run distance and bouton formation at 24hrs. However, the correlation does not reach significance (linear regression slope significance, p=0.2631). One male included in b did not have a 24hr imaging time point and thus is excluded in c. (**d**) 24hr Normalized microglial volume correlates with the AUC of the Sholl curve (n: control=10 wheel=12 linear regression slope significance, F(1,18)=7.696, p=0.0125) (**e**) 24hr Normalized microglial volume correlates with the maximum intersections of the Sholl curve (n: control=10, wheel=12; linear regression slope significance, F(1,18)=4.927, p=0.0395). Graph shows mean ± S.E.M. Individual points represent individual animals with females as hollow symbols and males as solid symbols. Source data are provided as a source data file.

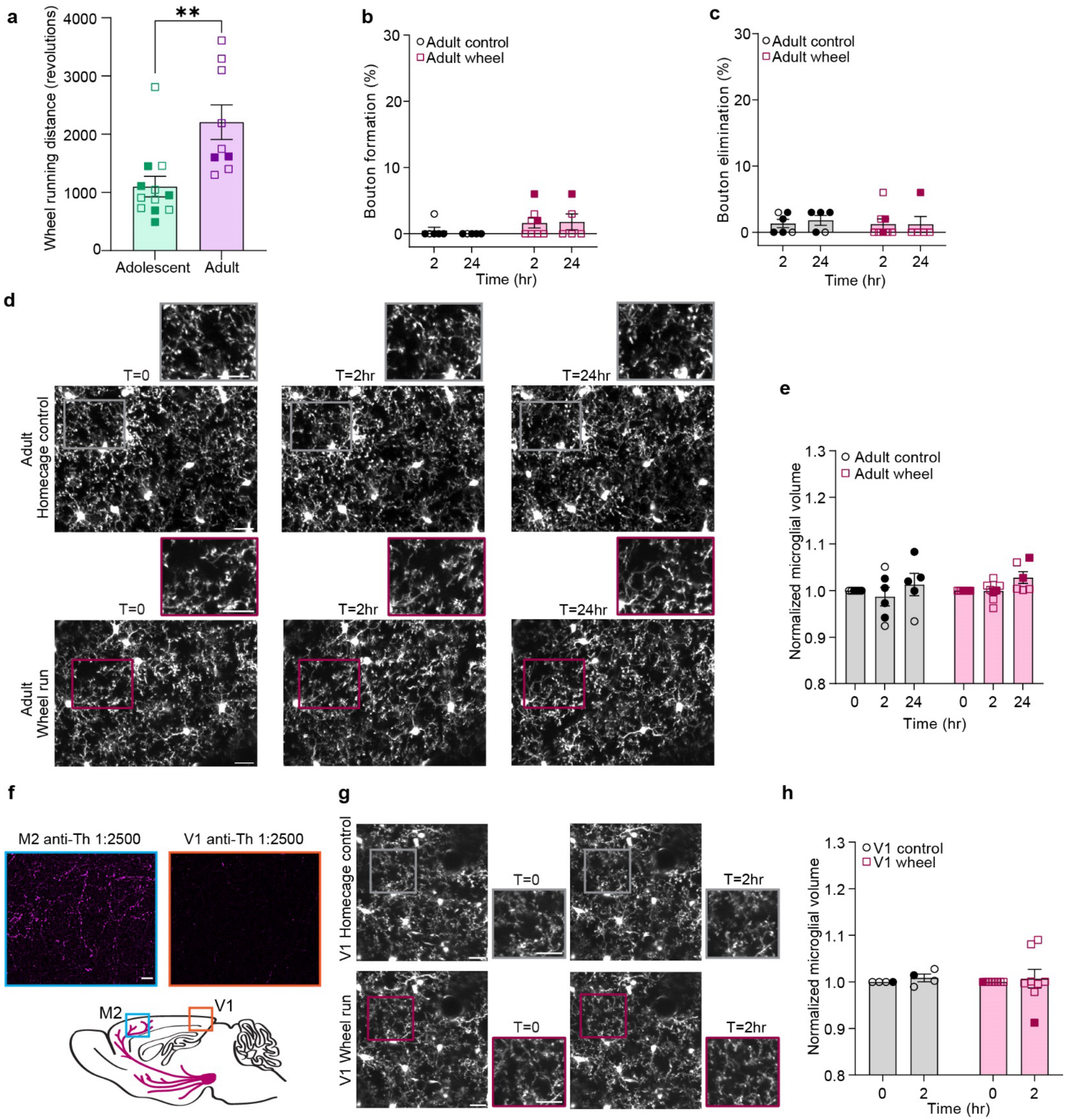

Supplementary figure 3| DA bouton outgrowth and the concomitant microglial process outgrowth are unique to the adolescent frontal cortex. (**a**) Adult mice run more than adolescent mice during 2hrs on a running wheel (n: adolescent=12, adult=9 mice, two-sided unpaired t-test p=0.0032, t(19)=3.375).(**b**) Wheel running does not drive DA bouton outgrowth in adult mice (n: control=6, wheel=8 mice, Mixed-effects model, Fixed effects [type III], Run status p=0.1443, F(1,12)=2.440, Šídák's multiple comparisons Control v. Wheel 2hr p=0.25 & 24hr p=0.25).(**c**) Wheel running does not drive DA bouton elimination in adult mice (n: control=6, wheel=8 mice, Mixed-effects model, Fixed effects [type III], Run status p=0.69, F(1,20)=0.1638, Šídák's multiple comparisons Control v. Wheel 2hr p=0.9964 & 24hr p=0.8742).(**d**) Example 30µm z-projections of adult microglia in the M2 frontal cortex. Rectangle pop-outs denote representative areas of microglial process volume. (**e**) Wheel running does not increase microglial volume in adult mice (n: control=6, wheel=8 mice, Mixed-effects model, Fixed effects [type III], Run status p=0.4076, F(1,12)=0.7364, Šídák's multiple comparisons Wheel 0 v. 2hr p=0.9786 & 0 v.

24hr p=0.1115) (**f**) Anti-tyrosine hydroxylase staining of M2 and V1 at a 1:2500 dilution, which captured dopaminergic but not noradrenergic fibers. Below the staining is a diagram highlighting the substantial dopaminergic innervation of M2 compared to V1, which receives few dopaminergic projections. (**g**) Example 30μm z-projections of microglia. Rectangle pop-outs denote representative areas of microglial process volume. (**h**) Wheel running does not increase microglial volume in V1 (n: control=4, wheel=6 mice, Two-way repeated measures ANOVA, p=0.9538, F(1,10)=0.003523). Scale bars 20μm. Multiple comparisons are two-sided. Graphs show mean ± S.E.M **p<0.01. Individual points represent individual animals with females as hollow symbols and males as solid symbols. Source data are provided as a source data file.

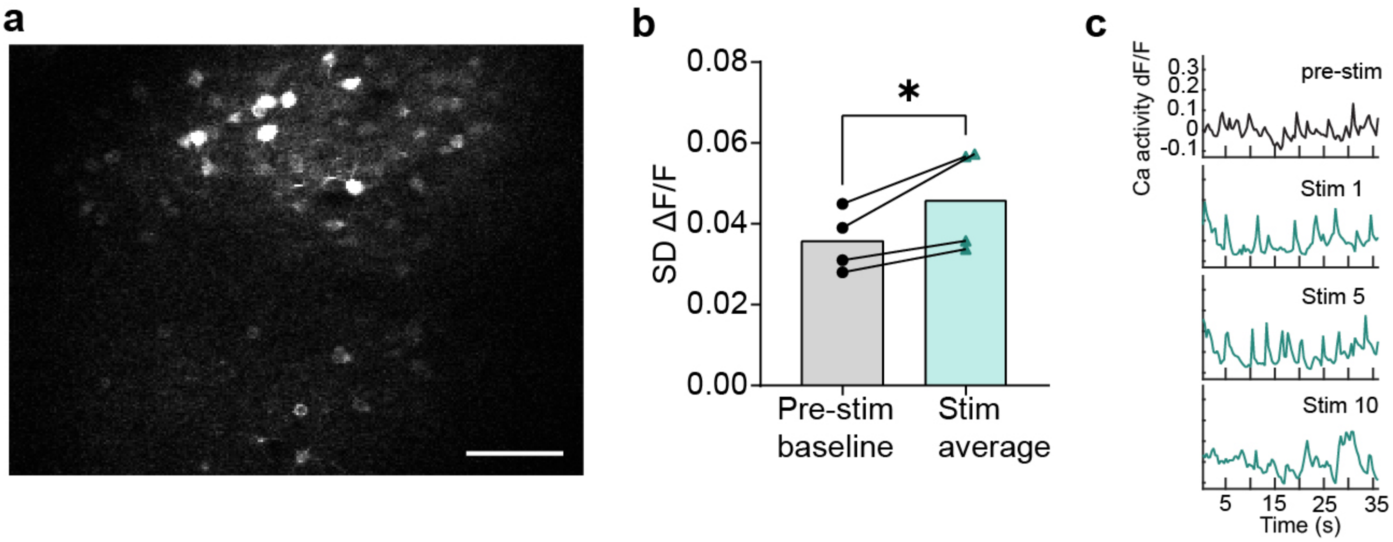

Supplemental Figure 4| Phasic optogenetic stimulation of the mesofrontal DA axons increases cortical neuronal activity. (**a**) Representative frame from an *in vivo* two-photon calcium imaging session. M2 frontal cortical neurons were labeled with AAV9-CAMKII-GCaMP6s injected locally. (**b**) Phasic optogenetic stimulation of the mesofrontal DA axons (473nm, 20mW output, 50Hz pulse train, 3ms/pulse, 10 pulses/train, 1 train/min for 10min) elicits a significant change in calcium activity in M2 neurons. Calcium imaging was conducted (0.367s/frame, 100 frames) just prior to stimulation (pre-stim baseline) and in between each of the ten pulses. The standard deviation (SD) of the calcium activity (dF/F) trace in those ten responses were averaged and compared to that in the pre-stim baseline. (n=4 mice, two-tailed paired t-test, p=0.0472, t(3)=3.259). (**c**) Example traces from just prior to stimulation (pre-stim) and between stimulation pulses. Scale bar 80μm. Graph shows mean ± S.E.M. Individual points represent individual animals. Source data are provided as a source data file.

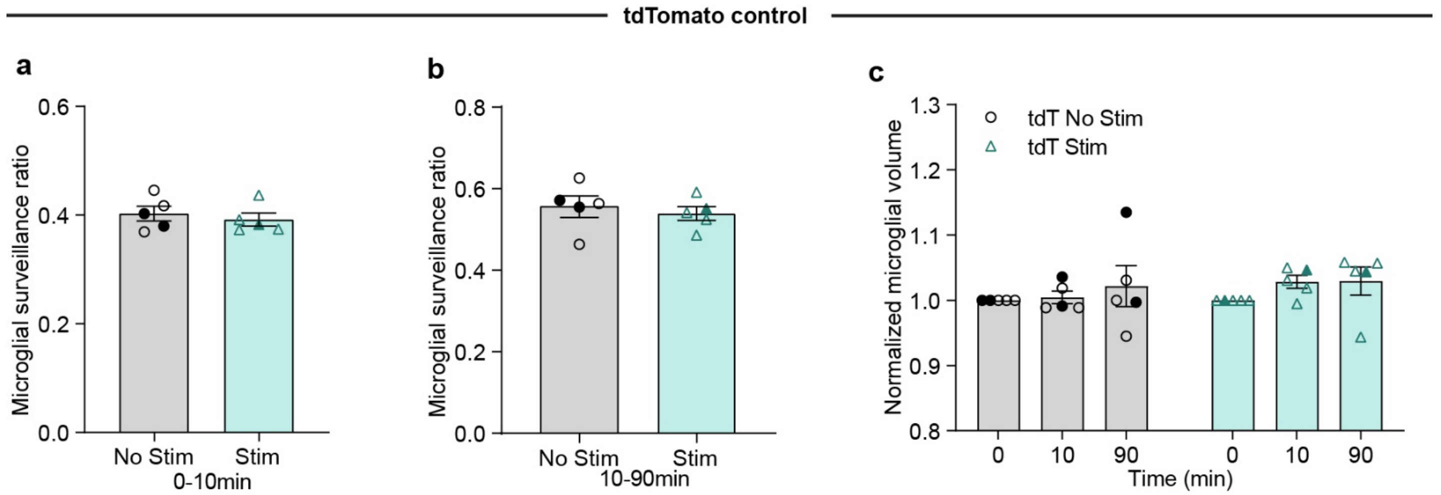

Supplemental Figure 5| In the absence of ChR2 expression, the phasic light stimulation paradigm does not alter adolescent microglial dynamics. (**a**) Microglial surveillance is unchanged during phasic light stimulation in the absence of ChR2 (n=5 mice, two-tailed unpaired t-test, p=0.5553, t(8)=0.6155). (**b**) Microglial surveillance is unchanged after phasic light stimulation in the absence of ChR2 (n=5 mice, two-tailed unpaired t-test, p=0.6068, t(8)=0.5355). (**c**) Microglial volume is not altered by phasic light stimulation in the absence of ChR2 (n=5 mice, two-way repeated measures ANOVA, time x stimulation p=0.7867, F(2,16)=0.2436). Graphs show mean ± S.E.M. No Stim and Stim experiments were conducted within the same animals. Individual points represent individual animals with females as hollow symbols and males as solid symbols. Source data are provided as a source data file.

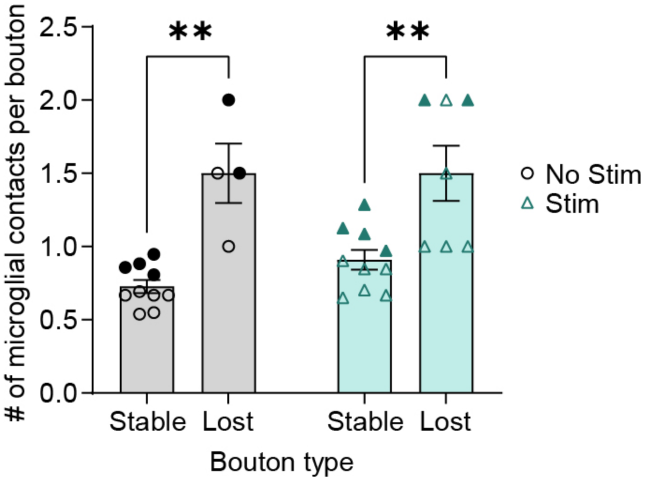

Supplemental Figure 6| In adolescents, microglia contact eliminated boutons more frequently than stable boutons. Eliminated boutons are contacted more frequently by microglia than stable boutons (Mixed-effects model, Fixed effects [type III], bouton type p=0.0007, F(1,16)=17.57, Holm-Šídák's multiple comparisons, 24hr, No Stim Stable v. Lost p=0.0010, Stim Stable v. Lost p=0.0012). Multiple comparisons are two-sided. Graph shows mean ± S.E.M. **p<0.01. No Stim and Sim experiments were conducted within the same animals. Individual points represent individual animals with females as hollow symbols and males as solid symbols. Source data are provided as a source data file.

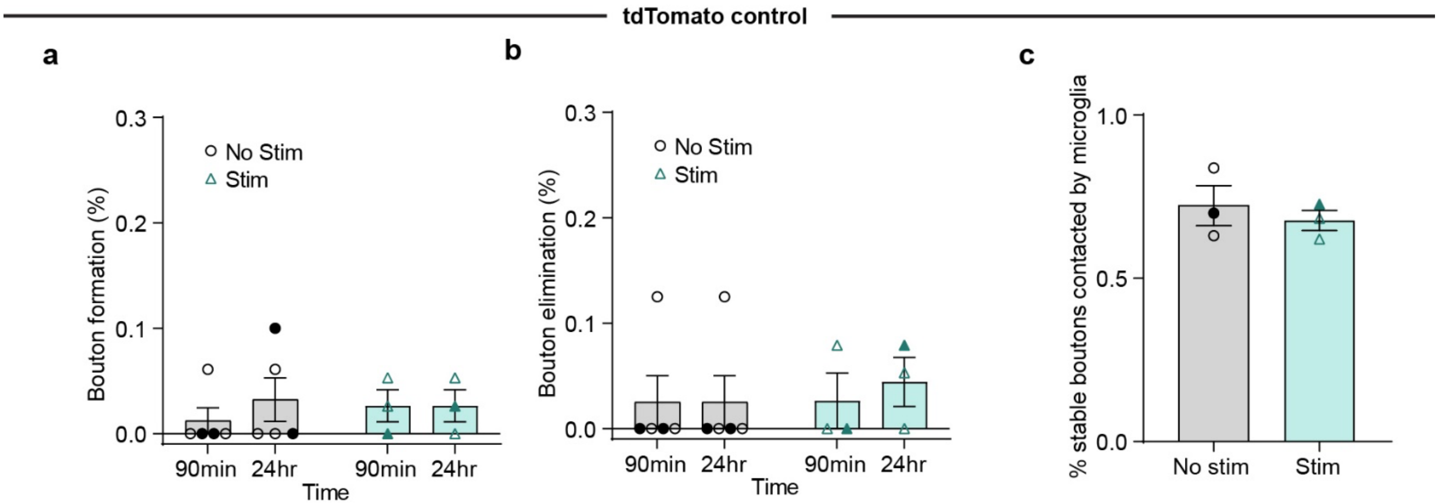

Supplemental Figure 7| In the absence of ChR2 expression, the phasic light stimulation paradigm does not alter adolescent mesofrontal plasticity or microglial dynamics. (**a**) Phasic light stimulation does not alter bouton formation rates in the absence of ChR2 (n=5 mice, two-way repeated measures ANOVA, time x stimulation p=0.5661, F(1,6)=0.3684). (**b**) In the absence of ChR2, phasic light stimulation does not alter bouton elimination rates (n=5 mice, two-way repeated measures ANOVA, time x stimulation P=0.6385, F (1, 6) = 0.2446) (**c**) In the absence of ChR2, microglial contacts with stable boutons are not altered by phasic light stimulation (n=3 mice, two-tailed unpaired t-test, p=0.5418, t(4)=0.6661) Graphs show mean ± S.E.M. No Stim and Stim experiments were conducted within the same animals. Individual points represent individual animals with females as hollow symbols and males as solid symbols. Source data are provided as a source data file.

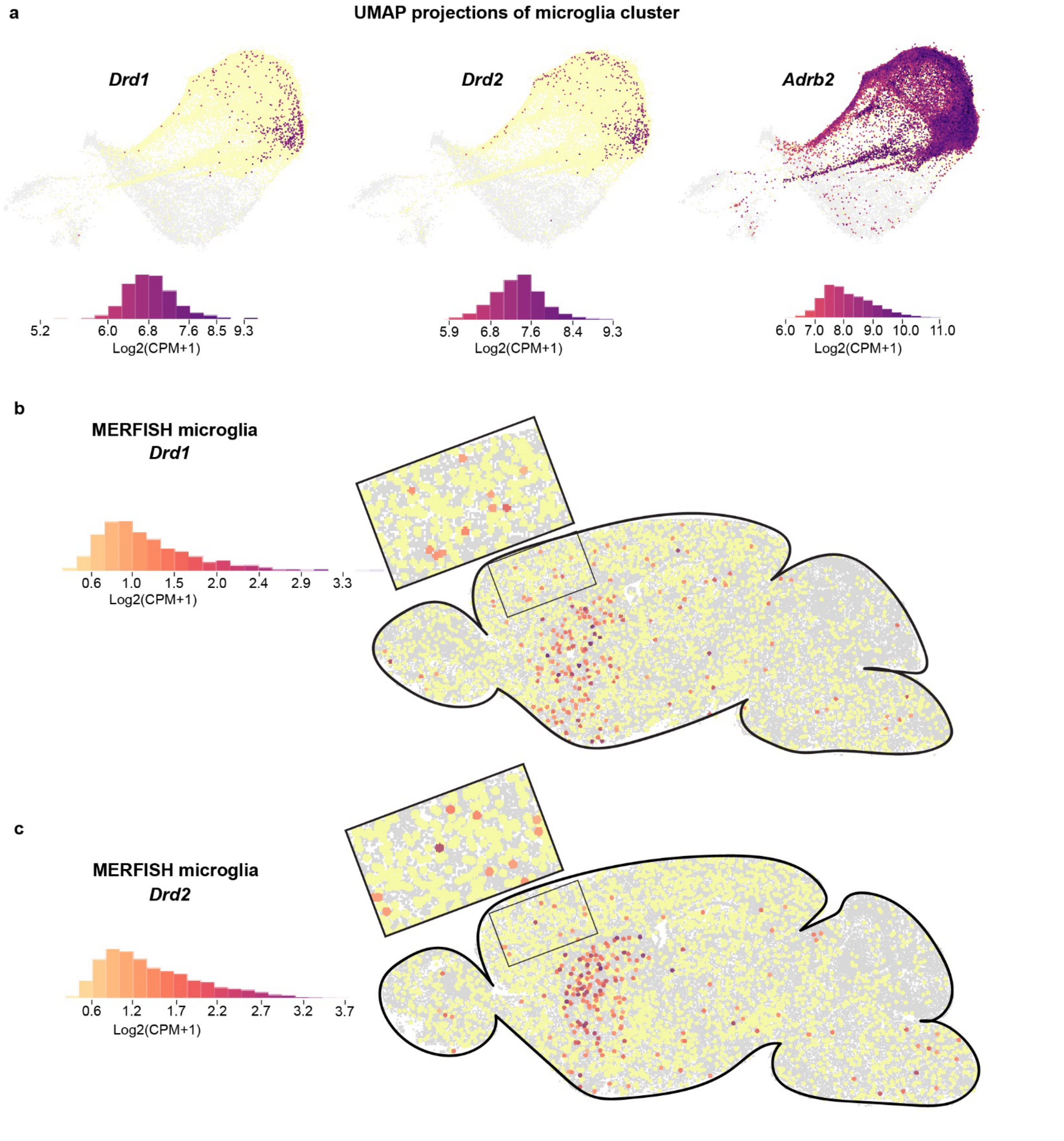

Supplemental Figure 8| Microglia express both *Drd1* and *Drd2*. Displayed data is adapted from the Allen Brain Atlas ABC tool (**a**) 10x scRNAseq UMAP of microglia cluster (pale yellow) labeled for expression (purple) of drd1, drd2, and adrb2. *Adrb2* is displayed for reference as it encodes a neurotransmitter receptor widely expressed by the microglial population, whereas *Drd1* and *Drd2* appear to have more selective patterns of expression. (**b**) Sagittal MERFISH for *Drd1* positive microglia (microglia are pale yellow, *Drd1*+ are coral and pink dots). (**c**) Sagittal MERFISH for *Drd2* positive microglia (microglia are pale yellow, *Drd2*+ are coral and pink dots).

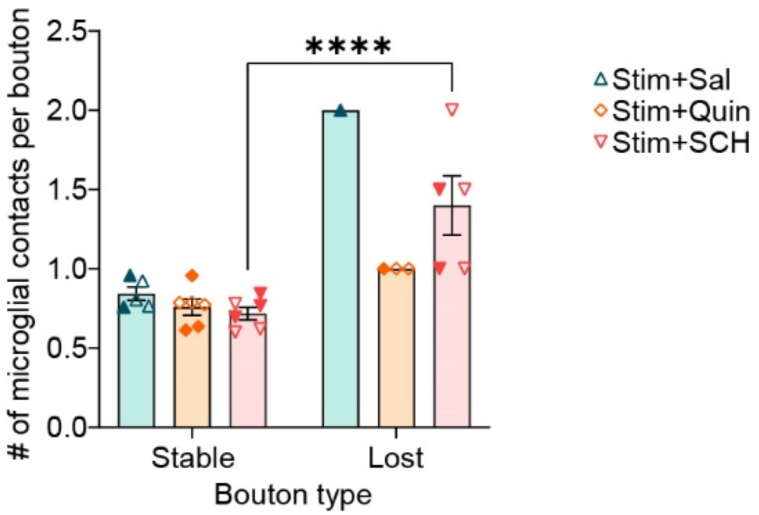

Supplemental Figure 9| Adolescent microglia make more contacts with eliminated boutons than stable boutons after optogenetic DA stimulation and D1 inhibition in adolescence. Mixed-effects model, Fixed effects [type III] interaction p=0.0079 F(2,20)=6.232, Šídák's multiple comparisons 24hr Stable v. Lost, Stim+SCH p<0.0001. Multiple comparisons are two-sided. Graph shows mean ± S.E.M, ****p<0.0001. Individual points represent individual animals with females as hollow symbols and males as solid symbols. Source data are provided as a source data file.

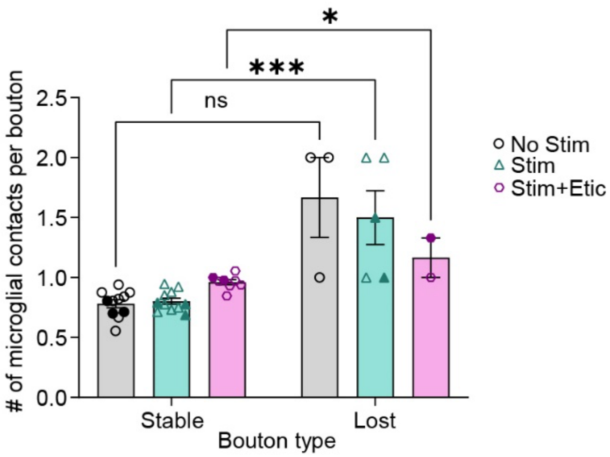

Supplemental Figure 10| In adults, microglia make more frequent contacts with eliminated boutons than stable boutons. Mixed-effects model, Fixed effects [type III] interaction $p<0.0001$ $F(2,17)=34.21$, Šídák's multiple comparisons 24hr Stable v. Lost: Stim $p=0.0004$, Stim+Etic $p<0.0312$. Multiple comparisons are two-sided. Graph shows mean ± S.E.M *$p<0.05$, ***$p<0.005$. No Stim, Stim and Stim+Etic experiments were conducted within the same animals. Individual points represent individual animals with females as hollow symbols and males as solid symbols. Source data are provided as a source data file.

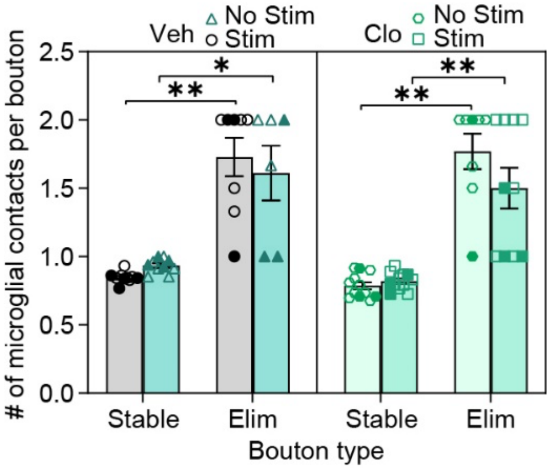

Supplemental Figure 11| With P2RY12 inhibition, microglia continue to make more contacts with eliminated boutons than stable boutons in adolescence. Eliminated boutons receive more microglial contacts than stable boutons (n: Veh=10, Clo=10, Mixed-effects model, Fixed effects [type III] Main effect of Bouton Type $p<0.0001$, $F(1,20)=86.69$, Šídák's multiple comparisons test Stable No Stim+Veh v. Elim No Stim+Veh $p=0.0034$ Stable Stim+Veh v. Elim Stim+Veh $p=0.0136$ Stable No Stim+Clo v. Elim No Stim+Clo $p=0.0013$ Stable Stim+Clo v. Elim Stim+Clo $p=0.0069$). Multiple comparisons are two-sided. Graph shows mean ± S.E.M *$p<0.05$, **$p<0.01$. No Stim+Veh and Stim+Veh experiments were conducted within the same animals. No Stim+Clo and Stim+Clo experiments were conducted within the same animals. Individual points represent individual animals with females as hollow symbols and males as solid symbols. Source data are provided as a source data file.

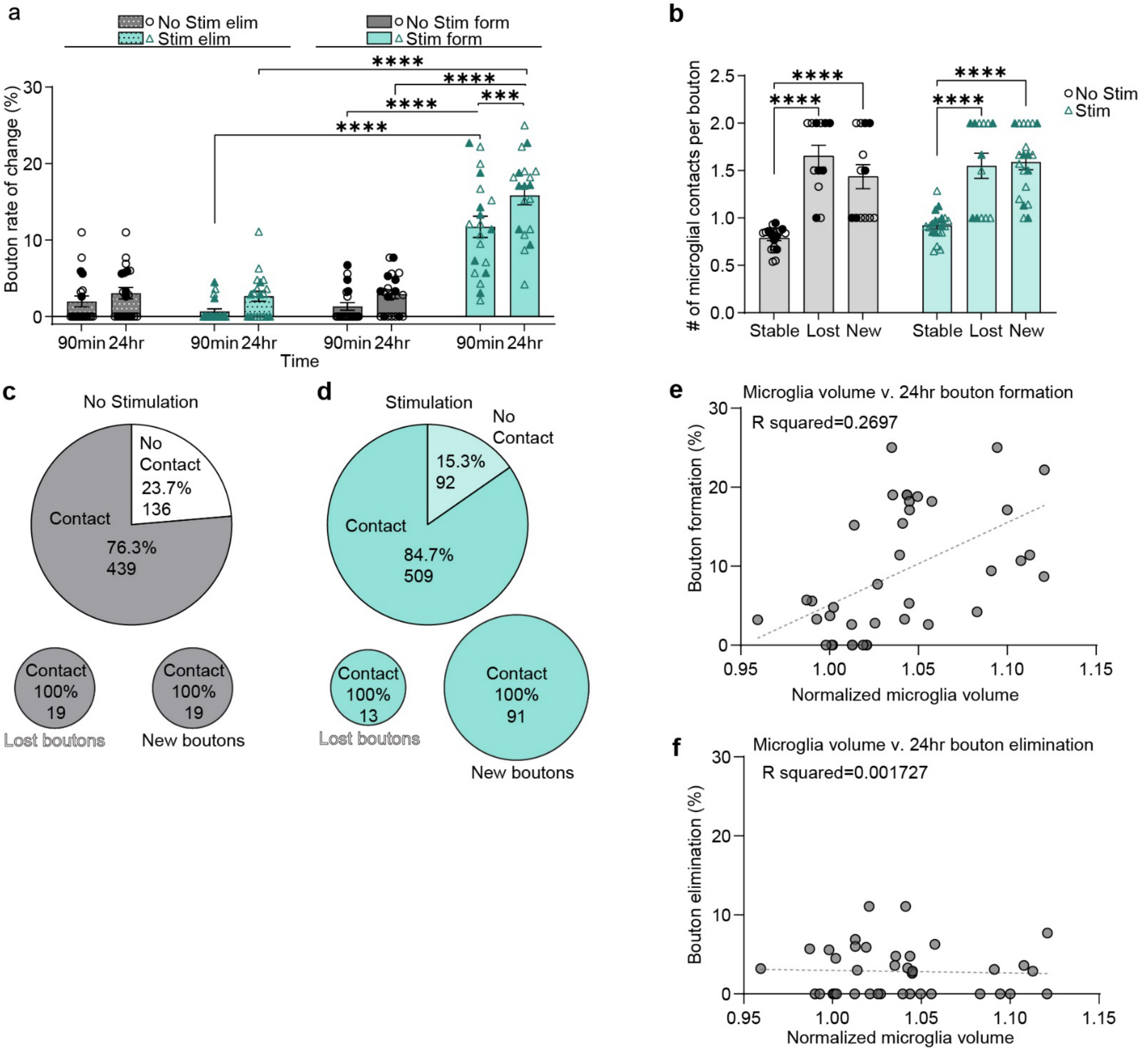

Supplemental Figure 12| Pooled optogenetic stimulation data show that although microglia are recruited to both newly formed and eliminated boutons, their activity is specifically predictive of bouton formation rates. (**a**) Phasic stimulation of DA axons drives bouton formation and not elimination (n=20 mice, Mixed-effects model Fixed effects [Type III] Stimulation x change p<0.0001, F(1,36)=82.30, Šídák's multiple comparisons Elim No stim v. Form Stim 90min p<0.0001, 24hr p<0.0001, Stim 90min v. 24hr p=0.0002 Elim Stim v. Form Stim 90min p<0.0001, 24hr p<0.0001).(**b**) Microglia make more frequent contact with new and eliminated boutons than stable boutons (n=20 mice, Mixed-effects model Fixed effects [Type III] Bouton type p<0.0001 F(2,38)=56.89 24hr No Stim Stable v. Lost p<0.0001, New p<0.001 Stim Stable v. Lost p<0.0001, New p<0.0001). (**c,d**) Pie charts showing pooled percentage (%) and number of stable, new boutons, lost boutons contacted by microglia in no stimulation (**c**) and stimulated (**d**) conditions. A higher % of stable boutons are contacted by microglia after stimulation. Both new boutons and lost boutons are always contacted by microglia, but only new boutons are more abundant after stimulation (illustrated by the size difference of the pie charts). (**e**) The microglial volume is correlated with bouton formation rates (n=20 mice, 39 values, Simple linear regression, significance of slope F(1,37)=13.66 p=0.0007). (**f**) The microglial volume is not correlated with bouton elimination rates (n=20 mice, 39 values, Simple linear regression, significance of slope F(1,37)=0.06402

p=0.8016). Multiple comparisons are two-sided. Graphs show mean ± S.E.M ***$p<0.005$, ****$p<0.0001$. Presented data is pooled from Figure 3 and Veh treated adolescent mice from Figure 8. No Stim and Stim experiments were conducted within the same animals. Individual points represent individual animals with females as hollow symbols and males as solid symbols. Source data are provided as a source data file.
