# Supplementary material for: Dopaminergic signaling regulates microglial surveillance and adolescent plasticity in the mouse frontal cortex
Source: Nat Commun. 2025 Aug 26;16:7974. doi: 10.1038/s41467-025-63314-4 (PMC12381247; doi:10.1038/s41467-025-63314-4)
Supplement: Supplementary file 2 — Description of Additional Supplementary Files [file 41467_2025_63314_MOESM2_ESM.pdf]

Description of Additional Supplementary Files

Supplemental Video 1

Time-lapse movie taken through a chronic cranial window showing the surveillance of adolescent microglia in the M2 frontal cortex of awake unstimulated mice taken over 10min at 1min intervals (10µm maximum intensity z-projections were compressed at each time point, video representative of observations made in n=11 mice) Scale bar, 20µm.

Supplemental Video 2

Time-lapse movie taken through a chronic cranial window showing the reduced surveillance of adolescent microglia in the M2 frontal cortex of awake mice in response to phasic optogenetic stimulation of DA axons taken over 10min at 1min intervals (10µm maximum intensity z-projections were compressed at each time point, video representative of observations made in n=11 mice) Scale bar, 20µm.

Supplemental Video 3

Time-lapse movie taken through a chronic cranial window showing the surveillance of adolescent microglia in the M2 frontal cortex of awake unstimulated mice taken over 80min at 10min intervals (30µm maximum intensity z-projections were compressed at each time point, video representative of observations made in n=11 mice) Scale bar, 20µm.

Supplemental Video 4

Time-lapse movie taken through a chronic cranial window showing the increased surveillance of adolescent microglia in the M2 frontal cortex of awake mice after phasic optogenetic stimulation of DA axons taken over 80min at 10min intervals (30µm maximum intensity z-projections were compressed at each time point, video representative of observations made in n=11 mice) Scale bar, 20µm.

Supplemental Video 5

Time-lapse movie taken through a chronic cranial window showing putative contacts between microglial processes and the axon backbone in the M2 frontal cortex of awake adolescent mice in response to phasic optogenetic stimulation of DA axons taken over 90min at 10min intervals and including the final 24hr time point (5µm maximum intensity z-projections presented to assist in visualizing contacts though analysis was conducted on individual slices, video representative of observations made in n=10 mice) Box highlights region of interest. Scale bar, 20µm.

Supplemental Video 6

Time-lapse movie taken through a chronic cranial window showing the reduced surveillance of adolescent microglia in the M2 frontal cortex of awake control mice dosed with Sal and receiving phasic optogenetic stimulation of DA axons taken over 10min at 1min intervals (10µm maximum intensity z-projections were compressed at each time point, video representative of observations made in n= 5 mice) Scale bar, 20µm.

Supplemental Video 7

Time-lapse movie taken through a chronic cranial window showing the reduced surveillance of adolescent microglia in the M2 frontal cortex of awake Quin dosed mice receiving phasic optogenetic stimulation of DA axons taken over 10min at 1min intervals (10µm maximum intensity z-projections were compressed at each time point, video representative of observations made in n= 6 mice) Scale bar, 20µm.

Supplemental Video 8

Time-lapse movie taken through a chronic cranial window showing that SCH inhibits adolescent microglial retraction in response to phasic optogenetic stimulation of DA axons taken over 10min at 1min intervals (10µm maximum intensity z-projections were compressed at each time point, video representative of observations made in n= 6 mice) Scale bar, 20µm.

Supplemental Video 9

Time-lapse movie taken through a chronic cranial window showing the increased surveillance of adolescent Sal dosed microglia in the M2 frontal cortex of awake mice after phasic optogenetic stimulation of DA axons taken over 80min at 10min intervals (30µm maximum intensity z-projections were compressed at each time point, video representative of observations made in n=5 mice) Scale bar, 20µm.

Supplemental Video 10

Time-lapse movie taken through a chronic cranial window showing that Quin prevents the increased surveillance of adolescent microglia in the M2 frontal cortex of awake mice after phasic optogenetic stimulation of DA axons taken over 80min at 10min intervals (30µm maximum intensity z-projections were compressed at each time point, video representative of observations made in n=6 mice) Scale bar, 20µm.

Supplemental Video 11

Time-lapse movie taken through a chronic cranial window showing that SCH dosing maintains microglia in a high state of surveillance in the M2 frontal cortex of awake adolescent mice after phasic optogenetic stimulation of DA axons taken over 80min at 10min intervals (30µm maximum intensity z-projections were compressed at each time point, video representative of observations made in n=6 mice) Scale bar, 20µm.

Supplemental Video 12

Time-lapse movie taken through a chronic cranial window showing the surveillance of adult microglia in the M2 frontal cortex of awake unstimulated mice taken over 10min at 1min intervals (10µm maximum intensity z-projections were compressed at each time point, video representative of observations made in n=11 mice) Scale bar, 20µm.

Supplemental Video 13

Time-lapse movie taken through a chronic cranial window showing the reduced surveillance of adult microglia in the M2 frontal cortex of awake mice in response to phasic optogenetic stimulation of DA axons taken over 10min at 1min intervals (10µm maximum intensity z-projections were compressed at each time point, video representative of observations made in n= 11 mice) Scale bar, 20µm.

Supplemental Video 14

Time-lapse movie taken through a chronic cranial window showing the reduced surveillance of adult microglia in the M2 frontal cortex of awake Etic dosed mice in response to phasic optogenetic stimulation of DA axons taken over 10min at 1min intervals (10µm maximum intensity z-projections were compressed at each time point, video representative of observations made in n= 11 mice) Scale bar, 20µm.

Supplemental Video 15

Time-lapse movie taken through a chronic cranial window showing the surveillance of adult microglia in the M2 frontal cortex of awake unstimulated mice taken over 80min at 10min intervals (30µm maximum intensity z-projections were compressed at each time point, video representative of observations made in n=11 mice) Scale bar, 20µm.

Supplemental Video 16

Time-lapse movie taken through a chronic cranial window showing the recovery of surveillance post-stimulation in adult microglia in the M2 frontal cortex of awake mice after phasic optogenetic stimulation of DA axons taken over 80min at 10min intervals (30µm maximum intensity z-projections were compressed at each time point, video representative of observations made in n=11 mice) Scale bar, 20µm.

Supplemental Video 17

Time-lapse movie taken through a chronic cranial window showing the increased surveillance of adult Etic dosed microglia in the M2 frontal cortex of awake mice after phasic optogenetic stimulation of DA axons taken over 80min at 10min intervals (30µm maximum intensity z-projections were compressed at each time point, video representative of observations made in n=9 mice) Scale bar, 20µm.

Supplemental Video 18

Time-lapse movie taken through a chronic cranial window showing the reduced surveillance of adolescent microglia in the M2 frontal cortex of awake Clo dosed mice receiving phasic optogenetic stimulation of DA axons taken over 10min at 1min intervals (10µm maximum intensity z-projections were compressed at each time point, video representative of observations made in n= 10 mice) Scale bar, 20µm.

Supplemental Video 19

Time-lapse movie taken through a chronic cranial window showing that Clo prevents the increased surveillance of adolescent microglia in the M2 frontal cortex of awake mice after phasic optogenetic stimulation of DA axons taken over 80min at 10min intervals (30µm maximum intensity z-projections were compressed at each time point, video representative of observations made in n=10 mice) Scale bar, 20µm.
