# Supplementary material for: Dopaminergic signaling regulates microglial surveillance and adolescent plasticity in the mouse frontal cortex
Source: Nat Commun. 2025 Aug 26;16:7974. doi: 10.1038/s41467-025-63314-4 (PMC12381247; doi:10.1038/s41467-025-63314-4)
Supplement: Supplementary file 22 — Reporting Summary [file 41467_2025_63314_MOESM22_ESM.pdf]

nature portfolio

Corresponding author(s): Kuan Hong Wang

Last updated by author(s): Jul 4, 2025

Reporting Summary

Nature Portfolio wishes to improve the reproducibility of the work that we publish. This form provides structure for consistency and transparency in reporting. For further information on Nature Portfolio policies, see our Editorial Policies and the Editorial Policy Checklist.

Statistics

For all statistical analyses, confirm that the following items are present in the figure legend, table legend, main text, or Methods section.

n/a	Confirmed	
☐	☒	The exact sample size (*n*) for each experimental group/condition, given as a discrete number and unit of measurement
☐	☒	A statement on whether measurements were taken from distinct samples or whether the same sample was measured repeatedly
☐	☒	The statistical test(s) used AND whether they are one- or two-sided<br>*Only common tests should be described solely by name; describe more complex techniques in the Methods section.*
☐	☒	A description of all covariates tested
☐	☒	A description of any assumptions or corrections, such as tests of normality and adjustment for multiple comparisons
☐	☒	A full description of the statistical parameters including central tendency (e.g. means) or other basic estimates (e.g. regression coefficient) AND variation (e.g. standard deviation) or associated estimates of uncertainty (e.g. confidence intervals)
☐	☒	For null hypothesis testing, the test statistic (e.g. *F*, *t*, *r*) with confidence intervals, effect sizes, degrees of freedom and *P* value noted<br>*Give P values as exact values whenever suitable.*
☒	☐	For Bayesian analysis, information on the choice of priors and Markov chain Monte Carlo settings
☒	☐	For hierarchical and complex designs, identification of the appropriate level for tests and full reporting of outcomes
☒	☐	Estimates of effect sizes (e.g. Cohen's *d*, Pearson's *r*), indicating how they were calculated

Our web collection on statistics for biologists contains articles on many of the points above.

Software and code

Policy information about availability of computer code

Data collection	Fluoview (FV10-ASW version 4.02.04:05) was used to collect all two-photon imaging data.
Data analysis	ImageJ (FIJI) and included plugins for the program were used to process the two-photon data for analysis. Matlab (2022 version), was used for calcium imaging analysis. Custom scrips were written and are available on Github (https://github.com/RDStowell/WangLab-MatLabScripts). Prism version 10 was used for statistical analysis.

For manuscripts utilizing custom algorithms or software that are central to the research but not yet described in published literature, software must be made available to editors and reviewers. We strongly encourage code deposition in a community repository (e.g. GitHub). See the Nature Portfolio guidelines for submitting code & software for further information.

Data

Policy information about availability of data

All manuscripts must include a data availability statement. This statement should provide the following information, where applicable:

- Accession codes, unique identifiers, or web links for publicly available datasets
- A description of any restrictions on data availability
- For clinical datasets or third party data, please ensure that the statement adheres to our policy

All data and code used for analysis will be made available when requested from the corresponding author. All matlab code will also be made freely available on GitHub.

Research involving human participants, their data, or biological material

Policy information about studies with human participants or human data. See also policy information about sex, gender (identity/presentation), and sexual orientation and race, ethnicity and racism.

Reporting on sex and gender	N/A
Reporting on race, ethnicity, or other socially relevant groupings	N/A
Population characteristics	N/A
Recruitment	N/A
Ethics oversight	N/A

Note that full information on the approval of the study protocol must also be provided in the manuscript.

Field-specific reporting

Please select the one below that is the best fit for your research. If you are not sure, read the appropriate sections before making your selection.

- [x] Life sciences
- [ ] Behavioural & social sciences
- [ ] Ecological, evolutionary & environmental sciences

For a reference copy of the document with all sections, see nature.com/documents/nr-reporting-summary-flat.pdf

Life sciences study design

All studies must disclose on these points even when the disclosure is negative.

Sample size	Statistical methods were not used to pre-determine sample sizes, however our sample sizes follow the norms of the field and are similar to previously reported sizes from both our lab and other publications in the field. N's were also chosen to permit sex comparisons within our experimental results. (Stowell, R. D., Sipe, G. O., Dawes, R. P., Batchelor, H. N., Lordy, K. A., Whitelaw, B. S., ... & Majewska, A. K. (2019). Noradrenergic signaling in the wakeful state inhibits microglial surveillance and synaptic plasticity in the mouse visual cortex. Nature neuroscience, 22(11), 1782-1792.)
Data exclusions	Exclusion data was pre-established as the following: animals with injections that missed the VTA /had no viral construct expression were excluded from analysis.
Replication	All attempts at replication of the experiments were successful. Pilot experiments were done with small cohorts manipulating D1 and D2 signaling pharmacologically prior to the initial experiments. For all our experiments we started with small pilot cohorts prior to the full cohorts of animals. We saw all our effects replicated with repeated testing. Our optogenetic stimulation effects were repeated across the original cohort and the control group of the D1/D2 pharmacological experiments.
Randomization	Mouse litters were pseudo-randomized in assignment to experiments. Full randomization was not done as efforts were made to distribute both males and females into our groups.
Blinding	Investigators were blind to group allocation during all data analysis.

Reporting for specific materials, systems and methods

We require information from authors about some types of materials, experimental systems and methods used in many studies. Here, indicate whether each material, system or method listed is relevant to your study. If you are not sure if a list item applies to your research, read the appropriate section before selecting a response.

Materials & experimental systems

n/a	Involved in the study	
[ ]	[x]	Antibodies
[x]	[ ]	Eukaryotic cell lines
[x]	[ ]	Palaeontology and archaeology
[ ]	[x]	Animals and other organisms
[x]	[ ]	Clinical data
[x]	[ ]	Dual use research of concern
[x]	[ ]	Plants

Methods

n/a	Involved in the study	
[x]	[ ]	ChIP-seq
[x]	[ ]	Flow cytometry
[x]	[ ]	MRI-based neuroimaging

Antibodies

Antibodies used	Tyrosine hydroxylase rabbit anti-mouse (1:2500, EMD Millipore, AB152, Clone: L1003, lot#3638897).
Validation	The Th-antibody has been validated in our lab in previously published work and validation information is also available on the manufacturer's website. Both the existing literature and our internal repeated experiments confirm the efficacy of our protocols for anitbody dilution and use. (Mastwal, S., Ye, Y., Ren, M., Jimenez, D. V., Martinowich, K., Gerfen, C. R., & Wang, K. H. (2014). Phasic dopamine neuron activity elicits unique mesofrontal plasticity in adolescence. Journal of Neuroscience, 34(29), 9484-9496.)

Animals and other research organisms

Policy information about studies involving animals; ARRIVE guidelines recommended for reporting animal research, and Sex and Gender in Research

Laboratory animals	All mouse strains were on a C57BL/6 background and between ages P28-P52 (adolescent) and P70-P100 (adult). CX3CR1-GFP (JAX:005582) heterozygous mice were used to visualize microglia with in vivo two-photon imaging and were bred to ThCre mice (Gong, S. et al. Targeting Cre recombinase to specific neuron populations with bacterial artificial chromosome constructs. J Neurosci 27, 9817-9823 (2007). https://doi.org:10.1523/JNEUROSCI.2707-07.2007) to enable targeting of dopaminergic projections from the VTA to the frontal cortex. DAT-Cre (JAX: 006660) and Ai14 (JAX:007914) mice were also bred to CX3CR1-GFP mice in pilot experiments which ultimately deemed the mouselines not efficacious for our experimental aims.
Wild animals	This study did not use wild animals.
Reporting on sex	All experiments included both male and female mice. Mice were pseudo-randomly assigned to groups as efforts were made to balance male and female mice numbers across all groups and all experiments. In all of the figures, all individual data points are presented and female data points are hollow symbols and male data points are solid symbols. Sex was analyzed as a variable in the wheel running data (Figure 1c and Supplemental Figure 2b) due to the distribution of the male and female data in this experiment. Sex was not analyzed in additional experiments as no other apparent separation of data based on sex was present.
Field-collected samples	This study did not use field-collected samples.
Ethics oversight	The University of Rochester Committee on Animal Resources. Protocol 2018-035.

Note that full information on the approval of the study protocol must also be provided in the manuscript.

Plants

Seed stocks	N/A
Novel plant genotypes	N/A
Authentication	N/A
